# Secretion and assembly of functional mini-cellulosomes from synthetic chromosomal operons in *Clostridium acetobutylicum* ATCC 824

**DOI:** 10.1186/1754-6834-6-117

**Published:** 2013-08-20

**Authors:** Katalin Kovács, Benjamin J Willson, Katrin Schwarz, John T Heap, Adam Jackson, David N Bolam, Klaus Winzer, Nigel P Minton

**Affiliations:** 1 Clostridia Research Group, BBSRC Sustainable BioEnergy Centre, School of Life Sciences, Centre for Biomolecular Sciences, University of Nottingham, University Park, Nottingham, NG7 2RD, UK; 2 Institute for Cell and Molecular Biosciences, Newcastle University, Newcastle upon Tyne, NE2 4HH, UK; 3 Current address: Centre for Synthetic Biology and Innovation, Division of Molecular Biosciences, Imperial College London, South Kensington Campus, London, SW7 2AZ, UK

**Keywords:** Synthetic mini-cellulosomes, Consolidated bioprocessing, *Clostridium acetobutylicum*

## Abstract

**Background:**

Consolidated bioprocessing (CBP) is reliant on the simultaneous enzyme production, saccharification of biomass, and fermentation of released sugars into valuable products such as butanol. Clostridial species that produce butanol are, however, unable to grow on crystalline cellulose. In contrast, those saccharolytic species that produce predominantly ethanol, such as *Clostridium thermocellum* and *Clostridium cellulolyticum,* degrade crystalline cellulose with high efficiency due to their possession of a multienzyme complex termed the cellulosome. This has led to studies directed at endowing butanol-producing species with the genetic potential to produce a cellulosome, albeit by localising the necessary transgenes to unstable autonomous plasmids. Here we have explored the potential of our previously described Allele-Coupled Exchange (ACE) technology for creating strains of the butanol producing species *Clostridium acetobutylicum* in which the genes encoding the various cellulosome components are stably integrated into the genome.

**Results:**

We used BioBrick2 (BB2) standardised parts to assemble a range of synthetic genes encoding *C*. *thermocellum* cellulosomal scaffoldin proteins (CipA variants) and glycoside hydrolases (GHs, Cel8A, Cel9B, Cel48S and Cel9K) as well as synthetic cellulosomal operons that direct the synthesis of Cel8A, Cel9B and a truncated form of CipA. All synthetic genes and operons were integrated into the *C. acetobutylicum* genome using the recently developed ACE technology. Heterologous protein expression levels and mini-cellulosome self-assembly were assayed by western blot and native PAGE analysis.

**Conclusions:**

We demonstrate the successful expression, secretion and self-assembly of cellulosomal subunits by the recombinant *C. acetobutylicum* strains, providing a platform for the construction of novel cellulosomes.

## Background

The development of cost-effective strategies to synthesise biofuels from renewable biomass has received renewed interest from both academic and industrial institutions. Consolidated bioprocessing (CBP) is one such strategy, potentially allowing effective conversion of cheap lignocellulosic materials into high value industrial products [1]. This process requires simultaneous enzyme production, saccharification and fermentation of biomass into valuable products, without added enzymes. Current efforts to generate an organism suitable for CBP fall into two main categories, the ‘native’ method and the ‘recombinant’ method [2]; the former involves the engineering of existing cellulolytic organisms to display improved cellulose degradation and solvent production, whereas the latter involves the modification of an existing solvent producer to grow on lignocellulose. To date, most studies have focused on the production of ethanol (for an overview of developments in various organisms, see recent reviews by Olson et al. [2] and la Grange et al. [3]). Recent examples of engineering for ethanol production via CBP via the native method include the production of 38.1 g/l ethanol from 92.1 g/l Avicel via a co-culture of modified *Clostridium thermocellum* and *Thermoanaerobacterium saccharolyticum*[4]; the ‘recombinant’ approach has recently generated strains of *Saccharomyces cerevisiae*[5] and *Kluyveromyces marxianus*[6] able to ferment hot water treated rice straw and Avicel respectively, although final ethanol titres were still low.

Biobutanol has been recognised as an alternative transportation fuel to fossil fuels; its properties of a high calorific value, high hydrophobicity, low freezing point, and low heat of vaporisation make it a more attractive fuel than ethanol [7]. The best known solvent-producing bacterium is the mesophile *Clostridium acetobutylicum.* It efficiently converts all major monosaccharides generated from plant cell wall depolymerisation [8], and some polysaccharides like starch, into acids and solvents during the ABE (Acetone–Butanol–Ethanol) fermentation process [9,10]. In addition, *C. acetobutylicum* secretes xylan-degrading enzymes [11], although it is unable to utilise this polysaccharide efficiently [12]. Although some strains of *C. acetobutylicum* can grow on cellobiose and amorphous cellulose [11,12], they are unable to grow on crystalline cellulose [13], as the small amount of cellulosome produced appears to be non-functional in this organism. The introduction of a functional cellulosome is one potential approach to engineering *C. acetobutylicum* to be a suitable CBP organism.

Functional cellulosomes are large, multienzyme complexes designed for efficient deconstruction of cellulose and hemicellulose, the two most abundant polymers on Earth [14,15]. The core of the complex is a modular, non-catalytic scaffoldin protein which binds the cellulosome to its substrate via a non-catalytic carbohydrate binding module (CBM) and integrates the catalytic enzymatic subunits into the complex via cohesin-dockerin interactions (see recent review by Fontes and Gilbert [15]). One of the best characterised and most efficient cellulosomes is that of *C. thermocellum*. Moreover, *C. thermocellum* presents one of the highest rates of cellulose utilisation known, and its cellulosome displays good activity against crystalline cellulose [15], even at 37°C [16].

The *C. acetobutylicum* strain ATCC 824 has been previously used for heterologous production and secretion of individual cellulosomal components and mini-cellulosomes from *C. thermocellum* and *Clostridium cellulolyticum*[17-20]. In these studies, all cellulosomal subunits, cellulases and scaffoldin proteins were expressed from multi-copy plasmids, with their expression driven by either a weakened promoter or a promoter containing the *lac* operator, as cloning of the cellulolytic enzymes downstream of a strong promoter proved to be toxic to *Escherichia coli* cells [17-20]. In all of these systems, the levels of the secreted proteins, especially the larger family 9 and family 48 cellulases, were very low and the recombinant strains were unable to grow on crystalline cellulose. In the most recent study by Chanal et al. [20] the authors found that various scaffoldin modules, such as the family 3a carbohydrate binding module combined with the X2 modules from *C. cellulolyticum* or *C. acetobutylicum,* can trigger the secretion of larger cellulases when introduced to the cellulase as a carrier domain, but no further evidence for cellulose solubilisation was provided.

The expression of the cellulase genes from multi-copy plasmids raises potential issues regarding phenotype stability, especially if expression of the cellulases exerts a toxic effect on the cell as described by Mingardon et al. [19]. These issues were exemplified by the authors’ description of the generation of a heterologous *C. acetobutylicum* strain capable of expressing Cel48F from *C. cellulolyticum* with a subsequent loss of the phenotype after retrieval from spore stocks. Moreover, as no clostridial plasmids exhibit complete segregational stability in the absence of antibiotic selection, their use in a CBP process at industrial scale would be impractical.

Until recently, no suitable genetic tools have been available for constructing *C. acetobutylicum* strains containing heterologous DNA. The development of Allele-Coupled Exchange (ACE) [21] has made possible the generation of stable and iterative integrations within a comparatively short timescale. By using homology arms of different lengths, this approach allows control of the sequence of recombination events. At the second recombination event, a plasmid-borne sequence is coupled to a chromosomal sequence, forming a new allele that is selectable. Accordingly, in the present study we set out to test the potential of ACE for the construction of strains of *C. acetobutylicum* producing cellulosomes from gene clusters that are stably integrated into the genome, as opposed to being localised to unstable autonomous elements.

## Results

### Expression of *C. thermocellum* derived CipA scaffoldin variants in *C. acetobutylicum* driven by the host’s chromosomal thiolase promoter

**Figure 1:**
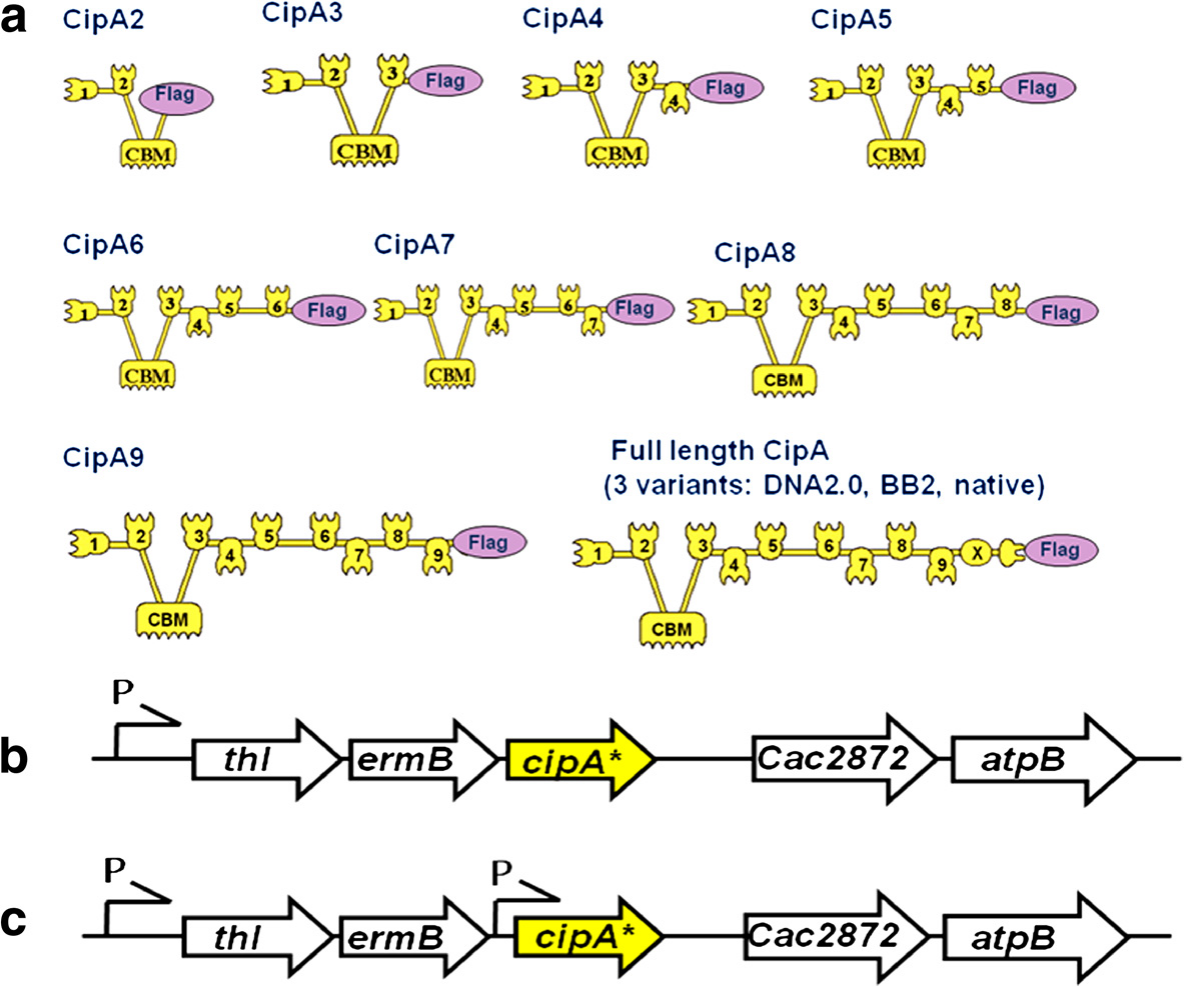
**Schematic representation of CipA variants integrated into the genome of *C. acetobutylicum *(a) and their integration sites into the chromosome without (b) and with (c) additional promoter (P).** All 11 CipA variants (CipA2-CipA full length) carry a C-terminal Flag tag. Encoded genes are: *thl*, thiolase; *ermB*, erythromycin ribosomal methylase B, cipA* variant, *CAC2872* predicted open reading frame and *atpB,* F0F1 ATP synthase subunit A.

CipA is the primary scaffoldin of *C. thermocellum* and is thus responsible for the integration of the enzymes into the complex [15,22]. To establish the maximum size of CipA and thus the complexity of the cellulosome that can be efficiently expressed and secreted by *C. acetobutylicum* various forms of the scaffoldin proteins were constructed; the modular nature of *C. thermocellum* CipA [Cthe_3077, NCBI: NC_009012] allowed the design and construction by standard BioBrick2 (BB2) assembly of synthetic modular parts of a series of scaffoldin proteins with different numbers of cohesin modules as well as the reconstruction of full length *cipA* (Figure 1a).

All CipA variants contained the native CipA endogenous signal peptide and the family 3a CBM (CBM3a), which binds to crystalline cellulose. The native full length *cipA* gene was PCR amplified from *C. thermocellum* genomic DNA and used to compare the expression level of the native gene to two full length codon optimised variants, one synthesised as a single fragment, the other built by BB2 assembly and therefore carrying several Ala-Ser substitutions (assembly “scars”) within the linker regions of the protein. All encoded CipA variants (synthetic and native) were fused at the carboxy-terminal with the Flag epitope tag, allowing the detection and quantification of the secreted scaffoldin proteins by western blot analysis.

All CipA variants were inserted into the *C. acetobutylicum* genome by Allele-Coupled Exchange (ACE) technology as described by Heap et al. [21], using the pMTL-JH16 integration plasmid. The CipA fragments were inserted downstream from the thiolase (*thl*) gene and upon integration were orientated to be under the transcriptional control of the strong, constitutive *thl* promoter (P_*thl*_) from the host chromosome (Figure 1b).

**Figure 2:**
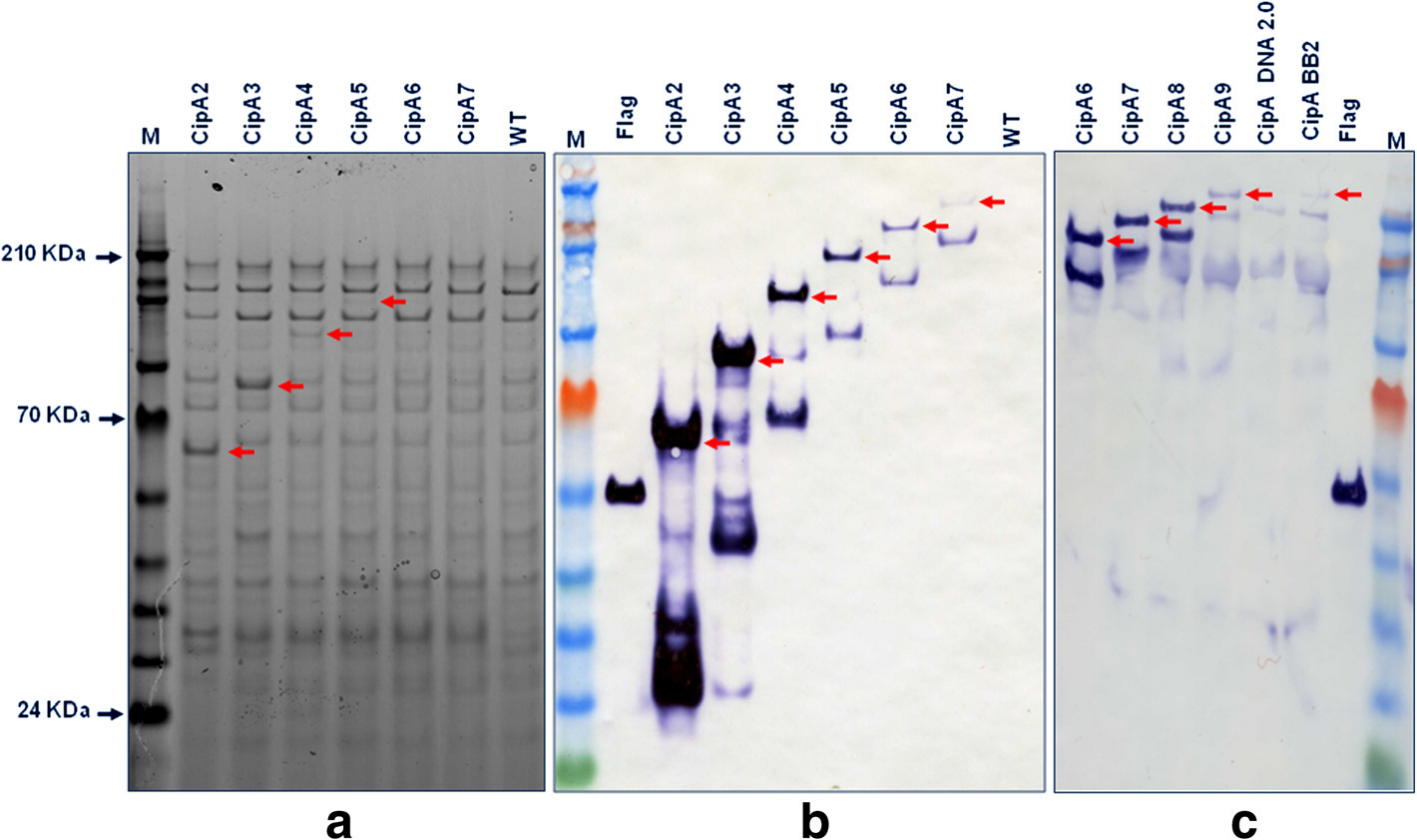
**SDS-PAGE and western blot analysis of all synthetic CipA variants.** Supernatants of CipA-expressing recombinant *C. acetobutylicum* strains were mixed with Avicel (crystalline cellulose) and proteins harbouring a carbohydrate binding module (CBM), including the CipA variants, were pulled down. **(a)** SDS-PAGE analysis and Simply blue staining (Invitrogen) of the Avicel bound culture supernatants of the first six CipA variants (CipA2-CipA7) **(b)** Western blot analysis of the Avicel bound CipA scaffold variants (CipA2-CipA7) using ANTI-FLAG M2 monoclonal antibody-horseradish peroxidise conjugate (Sigma). Samples were concentrated 100 fold. **(c)** Western blot analysis of the Avicel bound CipA scaffold variants (CipA6-CipA full length) using the same antibody. These samples were concentrated 150 fold. Expected molecular masses are indicated by red arrows. M, ColorPlus Prestained Protein Ladder (10–230 kDa; New England Biolabs), Flag, N-Flag-Bap control protein (50 kDa, 50 ng/lane; Sigma).

The chromosomal integration of all transgenes was confirmed by PCR and positive clones were selected and subjected to SDS-PAGE and FLAG® colorimetric western blot analysis. As each of the ten scaffoldin variants carries a CBM3a, the recombinant *C. acetobutylicum* culture supernatants were mixed with Avicel (crystalline cellulose) and proteins harbouring a carbohydrate binding module (CBM3a), including the heterologous CipA variants, were bound to Avicel (Figure 2). Avicel-bound proteins were recovered by boiling the samples in SDS sample buffer. This resulted in cleavage of the CipA scaffoldin proteins, due to a labile aspartyl-proline (Asp-Pro) peptide bond within the second cohesin domain which has been shown previously to be sensitive to cleavage upon boiling and in the presence of SDS [23].

**Figure 3:**
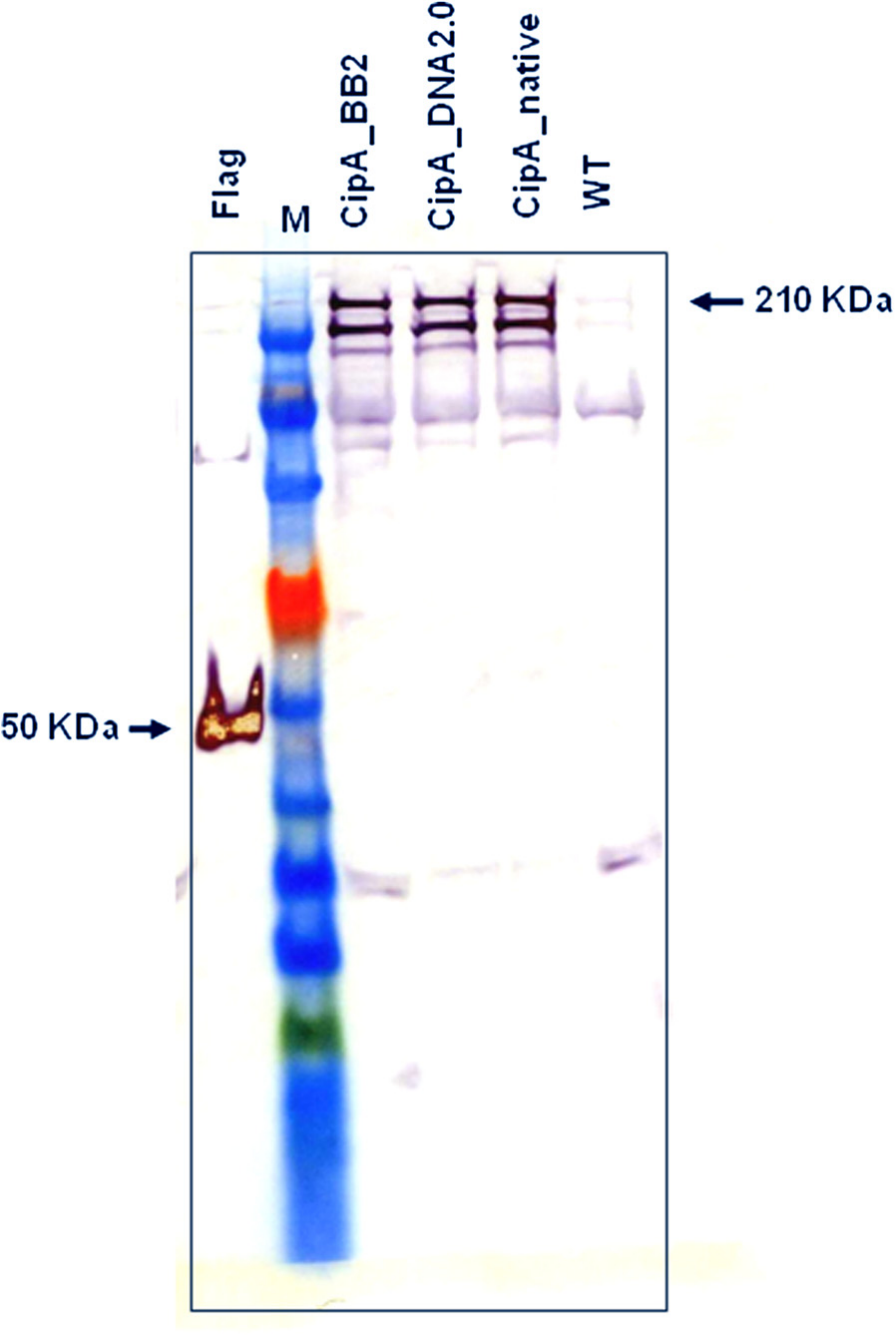
**Western blot analysis of the three full length CipA variants.** Supernatants of CipA-expressing recombinant *C. acetobutylicum* strains (CipA_BB2, CipA_DNA2.0 and CipAnative) were TCA precipitated and concentrated 200-fold. ANTI-FLAG M2 monoclonal antibody-horseradish peroxidise conjugate (Sigma) was used to detect the Flag-tagged CipA proteins. Expected molecular masses are indicated by red arrows. M, ColorPlus Prestained Protein Ladder (10–230 kDa; New England Biolabs), Flag, N-Flag-Bap control protein (50 kDa, 50 ng/lane; Sigma).

Western blot analysis indicated the successful expression and secretion of all CipA variants by the recombinant strains. Notably, the amounts of the secreted CipA variants were observed to decrease gradually with the addition of each cohesin domain. Using a Flag-tagged reference protein of known concentration (50 ng/lane) we estimated that the final concentration of cellulose bound CipA variants ranged from 0.001 mg/l to 0.1 mg/l. The intracellular levels of heterologous proteins were very low and almost identical in all samples (data not shown) suggesting that all the scaffoldin proteins were exported. The low yield of secreted scaffoldin proteins, compared to a previous report by Perret et al. [17], could be at least partially explained by the relatively lower gene copy number. Similar expression levels (at around 0.001 mg/l) were observed for all 3 full length CipA variants (Figure 3) indicating that neither codon optimisation nor the BB2 assembly scars had a substantial effect on the heterologous protein expression levels.

### Enhanced expression levels of CipA scaffoldin variants by using an additional promoter inserted downstream of the chromosomal promoter

**Figure 4:**
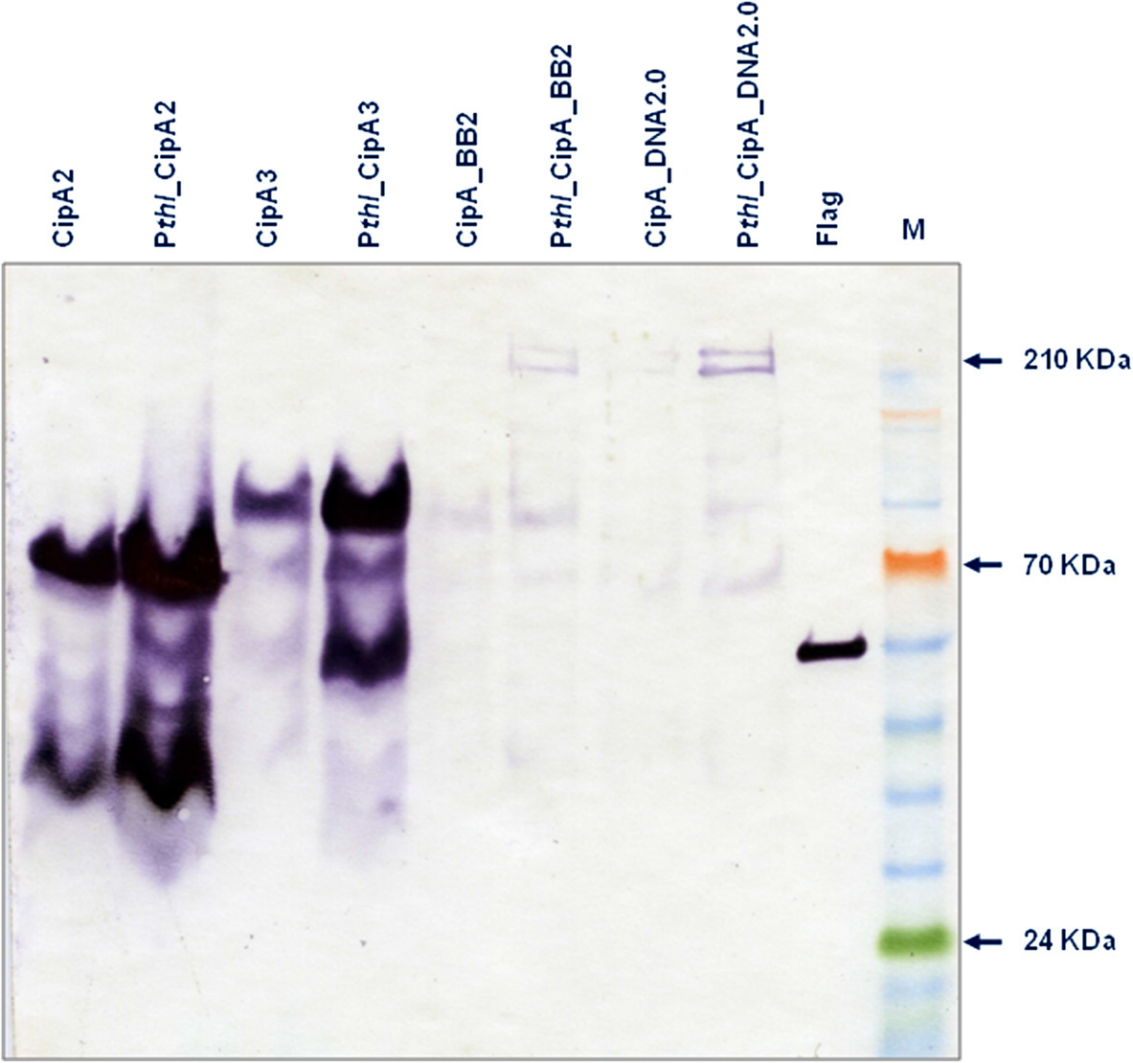
**Expression of CipA scaffoldin variants driven by an additional promoter.** Supernatants of CipA-expressing recombinant *C. acetobutylicum* strains (CipA2, CipA3, thlCipA2, thlCipA3, CipA_BB2, CipA_DNA2.0, thl_CipA_BB2, thl_CipA_DNA2.0) were TCA precipitated and concentrated 100-fold. ANTI-FLAG M2 monoclonal antibody-horseradish peroxidise conjugate (Sigma) was used to detect the Flag-tagged CipA variants. M, ColorPlus Prestained Protein Ladder (10–230 kDa; New England Biolabs). The N-Flag-Bap control protein (50 kDa, 50 μg/lane; Sigma) was used to estimate the amount of secreted CipA variants. Expected molecular masses are indicated by arrows.

Recombinant strains obtained by integrating heterologous genes downstream of the *thl* gene contain an artificial *thl*-*ermB*-inserted gene operon with the expression of all three genes dependent upon the chromosomal P_*thl*_ promoter (Figure 1b). This gene arrangement might not be ideal when high protein expression levels are desired, but allows the expression of heterologous genes which are lethal to *E. coli* cells when cloned downstream of a strong promoter. To test whether we could improve the expression levels of the scaffoldin variants, we inserted a second copy of the *thl* promoter upstream of the *cipA* coding regions; this insertion proved not to be lethal to *E. coli* and we were able to integrate the expression cassette into the same *thl* locus of *C. acetobutylicum*. (Figure 1c). We chose not to insert a transcriptional terminator upstream of the newly-inserted second promoter in order to maximise the number of *cipA* encoding transcripts by allowing read-through from the upstream native promoter. Western blot analysis indicated that the insertion of the additional *thl* promoter at least doubled the expression levels of the scaffoldin proteins (Figure 4), from around 0.1 mg/l to 0.2 mg/l for the shortest CipA variant. The effect of the additional promoter seemed to be equally substantial on the shorter scaffoldin variants (two or three cohesin modules) and the full length variants. In later experiments, the shorter scaffoldin variants (mini-scaffoldins) with two and three cohesin domains (CipA2 and CipA3) were chosen for co-expression with cellulolytic enzymes.

### Expression of *C. thermocellum*-derived cellulases in C. *acetobutylicum* driven by the host’s chromosomal thiolase promoter

After successful expression of the various scaffoldin units, we aimed to introduce *C. thermocellum*-derived cellulases into the *C. acetobutylicum* genome, individually as well as in combination with CipA scaffoldin subunits. Although we found that the presence of an additional promoter increased the expression levels of the heterologous scaffoldin variants, it was not possible to clone the cellulases behind the strong *thl* promoter as this proved to be lethal to *E. coli* cells. Therefore, the cellulases, with their endogenous signal sequences, were cloned with a ribosome binding sequence, but without a promoter, to prevent expression in *E. coli*. Upon insertion into the chromosome of *C. acetobutylicum*, transcription will be directed by the strong chromosomal *thl* promoter. Similarly, as we were unable to observe any benefit of codon optimisation on the expression level of the scaffoldin genes when compared to the native scaffoldin, we chose to express the native cellulase genes from *C. thermocellum*.

Four of the most abundant *C. thermocellum* cellulosomal cellulases [24,25], Cel8A (Cthe_0269), Cel9B (Cthe_0543), Cel48S (Cthe_2089) and Cel9K (Cthe_0412) were chosen for expression in *C. acetobutylicum* both individually and as an operon together with the shorter (two or three cohesin domain) CipA scaffoldin variants (mini-scaffoldins).

**Figure 5:**
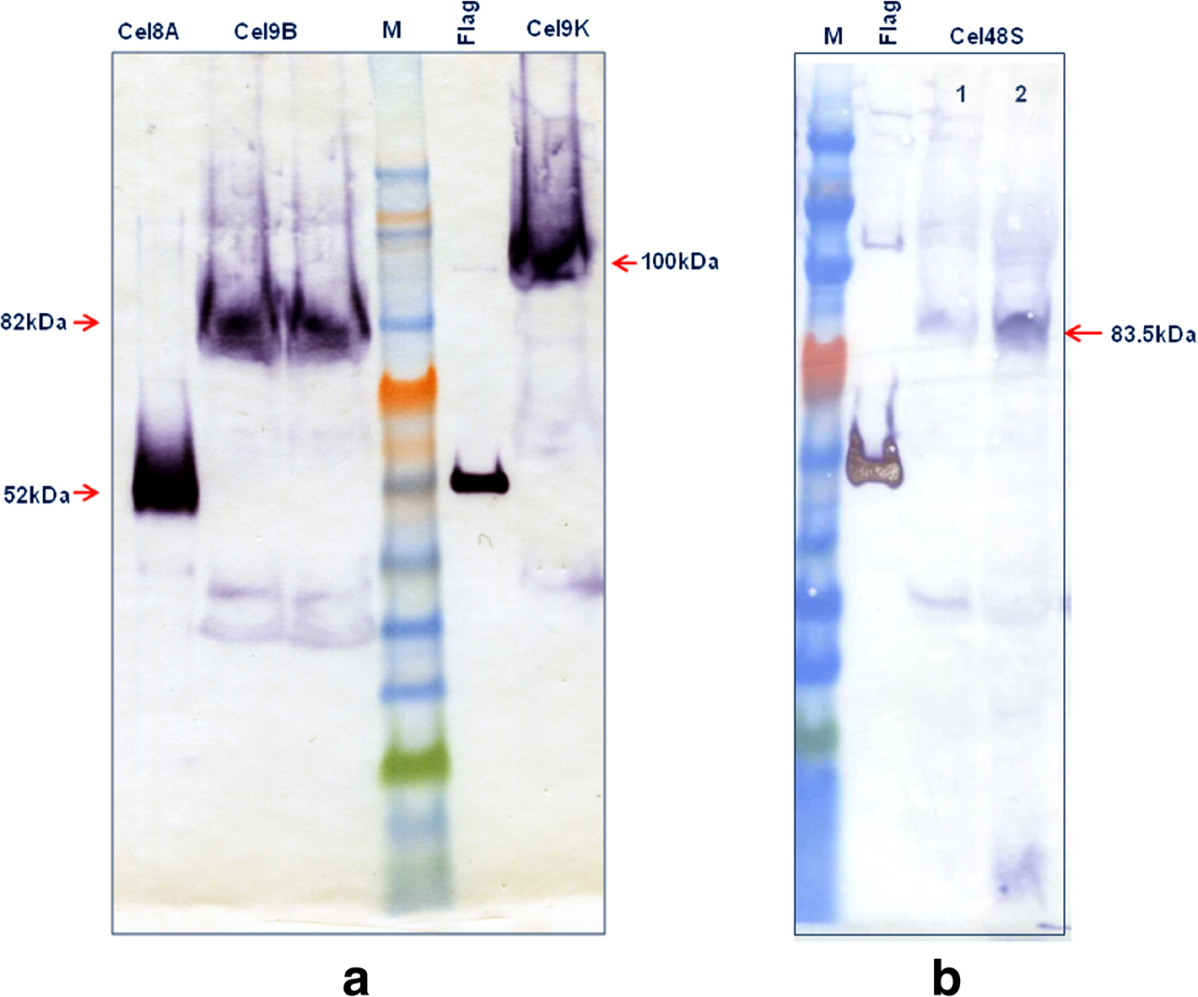
**Expression of *C. thermocellum *cellulases in *C. acetobutylicum. *(a)** Recombinant *C. acetobutylicum* strains expressing the Cel8A, Cel9B and Cel9K cellulases were grown until late stationary phase (OD600 > 3) and their supernatants TCA precipitated, concentrated 100-fold and analysed by SDS-PAGE. **(b)** The recombinant strain expressing the Cel48S was grown in buffered 2xYTG until early exponential phase (OD600 of 0.4[1] and 0.8[2]), concentrated 100-fold and separated by SDS-PAGE. All four cellulases were detected using ANTI-FLAG M2 monoclonal antibody-horseradish peroxidise conjugate (Sigma). Expected molecular masses are indicated by arrows. M, ColorPlus Prestained Protein Ladder (10–230 kDa; New England Biolabs), Flag, N-Flag-Bap control protein (50 kDa, 50 ng/lane; Sigma).

In order to confirm expression of the enzymes, western blot analysis was carried out on concentrated supernatants from recombinant *C. acetobutylicum* strains grown until late exponential phase (OD600 > 3), where the pH of the cultures dropped below 5 due to production of organic acids by the cells. Three (Cel8A, Cel9B and Cel9K) out of the four enzymes were expressed at similar but low levels (at around 0.05 mg/l) (Figure 5a), whereas Cel48S could not be detected at all. We observed that under these conditions, all other enzymes showed degradation products (Figure 5a), suggesting that Cel48S may be being completely degraded. In support of this, Cel48S could be detected in cultures grown in buffered media (pH 7, 40 mM MOPS) until early exponential phase (OD600 0.4-0.7) and before the pH of the culture dropped below 6 (Figure 5b). Degradation of heterologous cellulases by *C. acetobutylicum* has been previously reported, most often apparently due to proteolysis of the susceptible C-terminal dockerin domain [19,26,27]. Such truncations would not be detected in the experiments described here, as the Flag tag would be removed.

Secretion of the *C. cellulolyticum*-derived family 48 enzyme (Cel48F) and some larger family 9 (Cel9G and Cel9E) enzymes in *C acetobutylicum* has been previously shown to be difficult and it has been suggested that the secretion of these enzymes might require the presence of specific chaperone(s) not present in *C. acetobutylicum*[20].

### Expression of *C. thermocellum*-derived cellulosomal operons in *C. acetobutylicum*

To test whether our genome integration platform is suitable for expression of operons of various complexities from the chromosome of *C. acetobutylicum*, we built a series of cellulosomal operons comprising a mini-scaffoldin (CipA2 or CipA3) and one or two cellulases. For this, we chose Cel8A and Cel9B, to represent families containing small and large enzymes, respectively. Cel8A belongs to the family of GHs with a small catalytic domain shown to be secreted efficiently, both in previous studies [19] and in this work, while Cel9B belongs to the family of enzymes with large catalytic domains similar to Cel9G and Cel9E from *C. cellulolyticum*. In earlier studies, the expression of GH9 cellulases in *C. acetobutylicum* proved to be either toxic to the cells [19] or their secretion was only triggered when a CBM3a, combined with the X2 modules, was grafted at the N terminus of the protein [20]. Nevertheless, in the chromosomal system described above (see previous result section), Cel9B was expressed and secreted at similar level to Cel8A.

**Figure 6:**
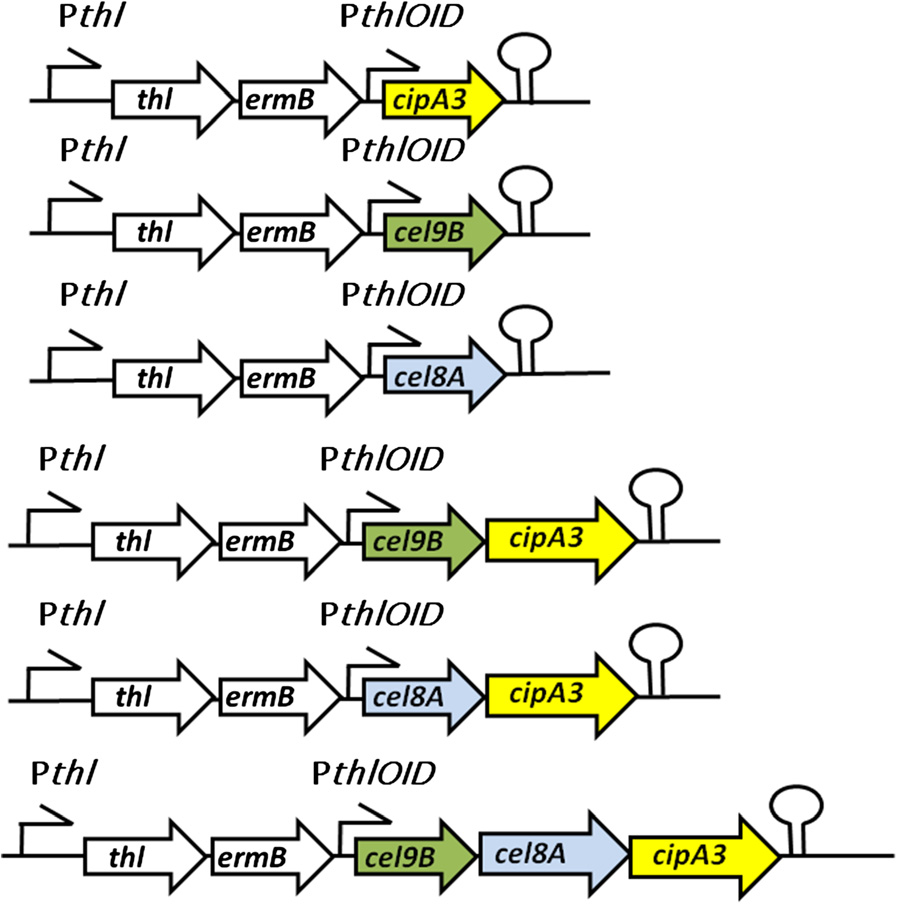
**Synthetic operons inserted into the *C. acetobutylicum *chromosome.** Encoded genes are: *thl*, thiolase; *ermB*, erythromycin ribosomal methylase B, *C. thermocellum* derived cellulases *cel9B* and *cel8A,* mini-scaffoldin *cipA3*. All operons are preceded by the chromosomal *thl* promoter and the celllulosomal components by an additional synthetic *thlOID* promoter and followed by a transcriptional terminator (from *Clostridium pasteurianum* ferredoxin gene).

In our initial experiments, we assembled operons containing the genes in the order they are found in simple cellulosomal systems, where the first gene of the operon is the scaffoldin gene followed by several hydrolytic enzymes. The first operons we tested contained a mini-scaffoldin (CipA2 or CipA3) and a hydrolase (Cel8A) integrated into the host’s thiolase locus and driven by the chromosomal thiolase promoter. We observed that both CipA2 and CipA3 mini-scaffoldin proteins were expressed at a higher level than Cel8A. In addition, the expression of Cel8A as part of the operon was lower than when it was expressed on its own (data not shown). These observations prompted us to make two alterations to our initial strategy. Firstly, as the mini-CipA3 gene displayed a greater level of expression than observed for Cel8A, we placed the mini-scaffoldin at the end of the operon, assuming that this would provide a better ratio of cellulase to scaffoldin. Secondly, as we had previously shown that adding an additional *thl* promoter leads to an increase in the level of expression of the scaffoldin variants, we placed all cellulases and cellulosomal operons under the control of an additional promoter. As it was not possible to clone any of the cellulase genes or cellulosomal operons downstream of the *thl* promoter in *E. coli*, we modified it to contain a symmetrical or ‘ideal’ *lac* operator sequence at its transcriptional start site (P*thl*OID promoter -nucleotide sequence can be found in Additional file 1), thereby allowing repression of transcription in an *E. coli* host harbouring *lacI*^*Q*^[17]. In this case, the *lacI*^*Q*^ gene was incorporated into the vector backbone. In total, six constructs containing the *thl*OID promoter were assembled in *E. coli* and subsequently integrated into the genome of *C. acetobutylicum* (Figure 6).

**Figure 7:**
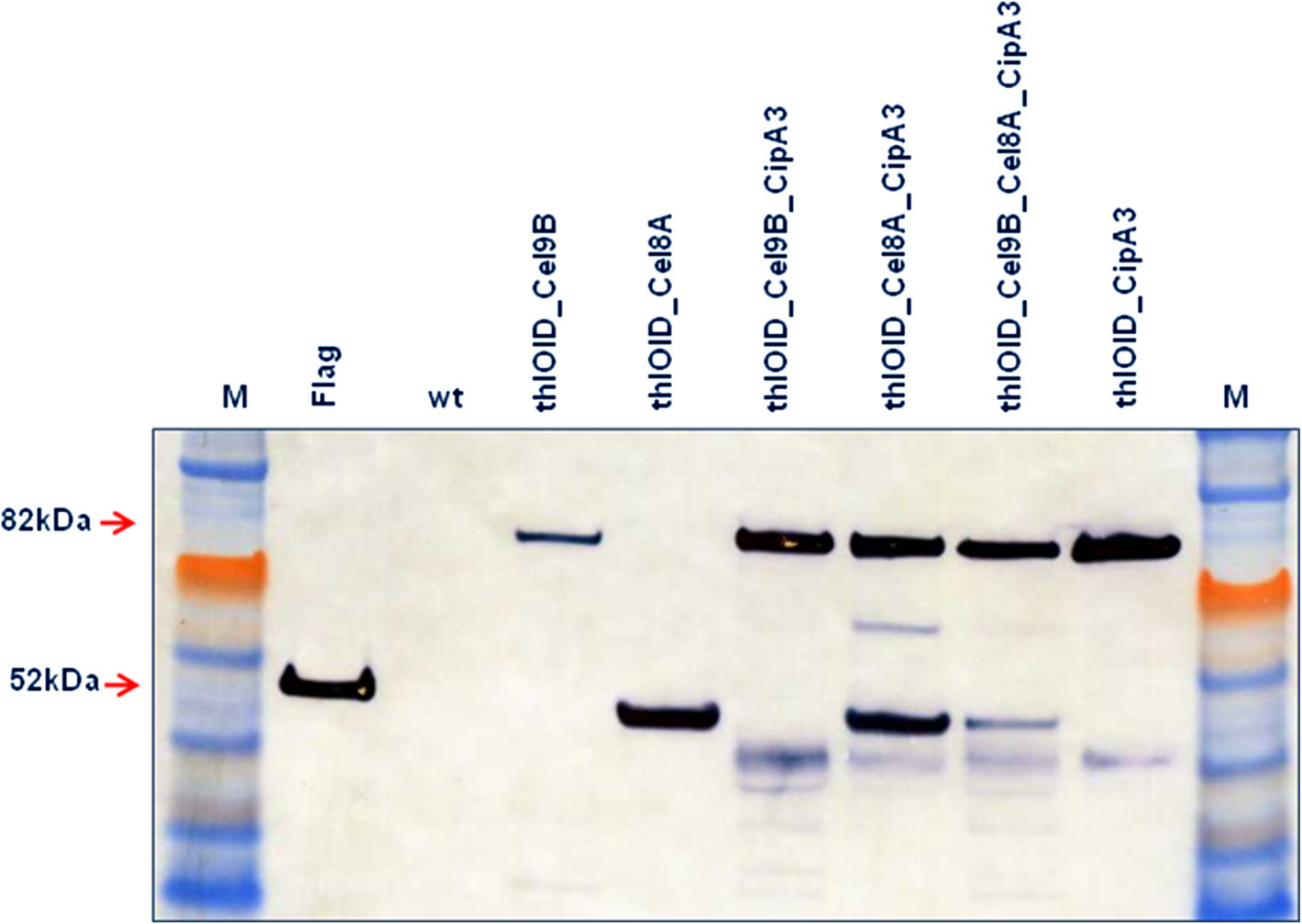
**Western blot analysis of cellulosomal operons secreted by the recombinant *C. acetobutylicum *strains.** The wild type (wt, *C. acetobutylicum* ATCC 824) and the recombinant strains (thlOIDCel9B, thlOIDCel8A, thlOIDCel9B_CipA3, thlOIDCel8A_CipA3, thlOIDCel9B_Cel8A_CipA3, thlOIDCipA3) were grown in buffered 2xYTG (pH 7 using 40 mM MOPS) until early exponential phase (OD600 0.8), concentrated 50-fold and separated by SDS-PAGE. All heterologous proteins were detected using ANTI-FLAG M2 monoclonal antibody-horseradish peroxidise conjugate (Sigma). M, ColorPlus Prestained Protein Ladder (10–230 kDa; New England Biolabs); Flag, N-Flag-Bap control protein (50 kDa, 50 ng/lane; Sigma). Expected molecular masses are indicated by arrows.

*C. acetobutylicum* recombinant strains expressing these cellulosomal operons were grown in buffered media (pH 7 using 40 mM MOPS) and tested at early exponential phase (OD600 around 0.7-0.8), before the pH of the cultures dropped below 6. Assaying the samples at early exponential phase ensured that the secreted heterologous proteins were intact; however their overall yield at the time of sampling was lower than expected (similar to that of strains expressing individual enzymes without an additional promoter, i.e. transcribed only from the upstream *thl* promoter, grown until late stationary phase). This is perhaps not surprising as the heterologous operons were placed under the control of a constitutively expressed promoter and the accumulation of secreted proteins is expected until late stationary phase. Western blot analysis (Figure 7) indicated that the expression levels of Cel8A (0.05 mg/l) and CipA3 (0.05 mg/l) were comparable when expressed as single entities, while Cel9B (0.01 mg/l) was synthesised at a much lower level. Furthermore, while the placement of *cel8A* before *cipA3* in the operon does not appear to decrease the expression of CipA3, it is apparent that positioning *cel9B* upstream of *cel8A* (or theoretically *cipA3* at the end of the three gene operon) has a significant negative impact on the expression of Cel8A. As Cel9B co-migrates with CipA3, we are unable to determine the level of expression of Cel9B or CipA3 from the western blot when these two proteins are co-expressed in the Cel9B-CipA3 or Cel9B-Cel8A-CipA operons.

### *In vivo* mini-cellulosome assembly by *Clostridium acetobutylicum*

**Figure 8:**
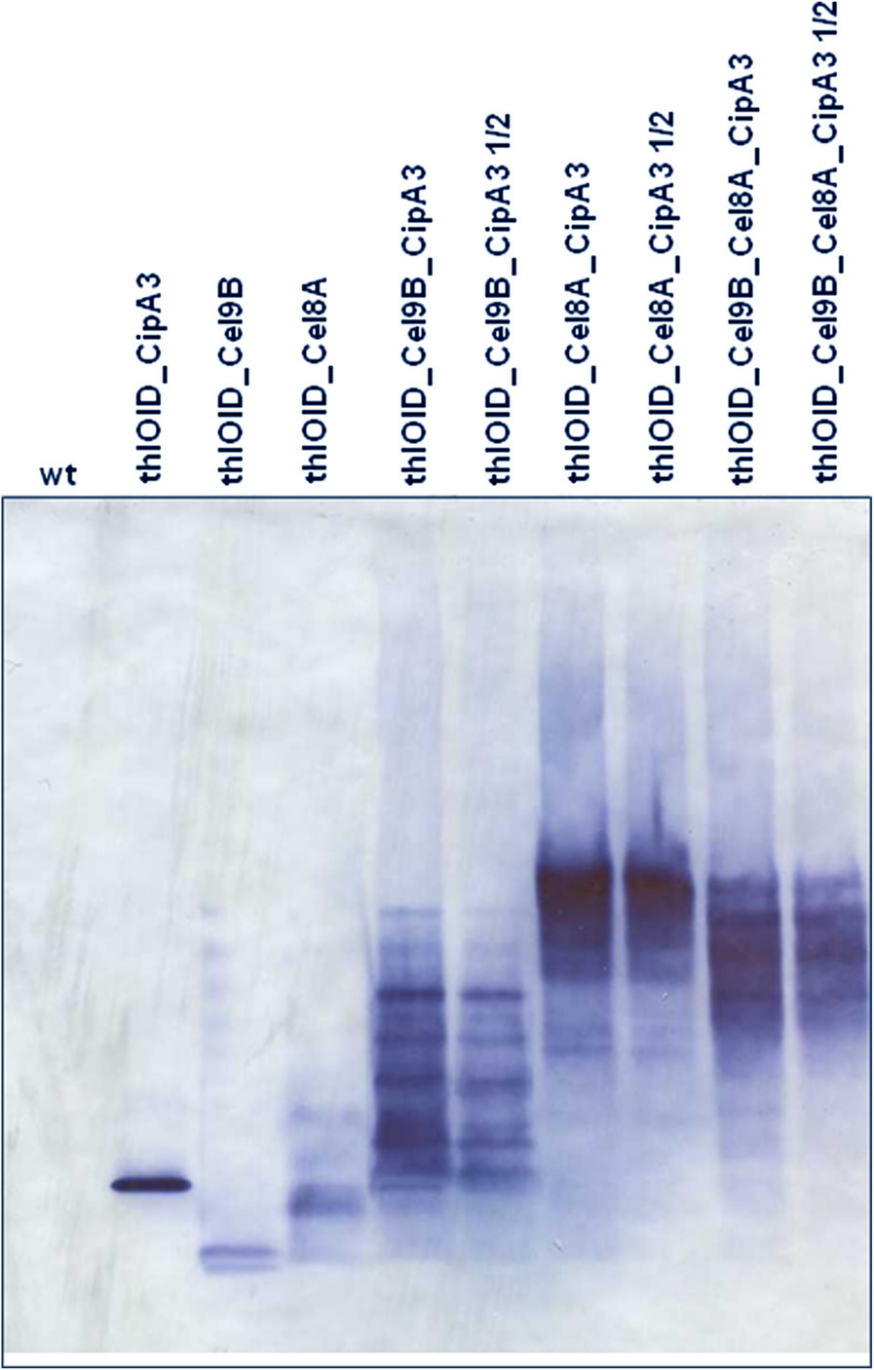
**Native PAGE and western blot analysis of cellulosomal operons assembled *in vivo *by the recombinant *C. acetobutylicum *strains.** The culture supernatants of the recombinant strains (thlOIDCel9B, thlOIDCel8A, thlOIDCel9B_CipA3, thlOIDCel8A_CipA3, thlOIDCel9B_Cel8A_CipA3, thlOIDCipA3) were concentrated 70-fold by ultracentrifugation and 10 μl of the concentrated samples loaded onto a native PAGE Tris-acetate gel. The presence of the cellulosomal subunits and the *in vivo* assembly of the mini-cellulosomes was detected using ANTI-FLAG M2 monoclonal antibody-horseradish peroxidise conjugate (Sigma). The presence of multiple bands in the samples expressing mini-cellulosomes (thlOIDCel9B_CipA3, thlOIDCel8A_CipA3 and thlOIDCel9B_Cel8A_CipA3) likely represents unsaturated complexes with cellulases binding different cohesin domains. These samples were loaded at two different concentrations: 10 and 5 μl (½) of the original samples.

In order to confirm the formation of a mini-cellulosome *in vivo*, we carried out native PAGE analysis on samples concentrated by ultrafiltration. Figure 8 shows that the cellulosomal components readily form complexes; the predominant rapid migrating species of CipA3, Cel8A and Cel9B are absent in the lanes of culture supernatants containing mixtures of the scaffoldin variant and the enzymes. The multiple bands likely reflects different isoforms of the enzymes (different charged species through deamidation of aspartates and glutamates or weak oligomerisation), although the complex pattern may also reflect incomplete saturation of CipA3. Additionally, it is possible to contrast the complexes formed by the expression of the three different operons; the complexes formed by Cel9B in combination with CipA3 display faster migration than those formed by Cel8A in combination with CipA3, while the co-expression of all three subunits generates a pattern that is a mixture of the two banding patterns. This latter hybrid pattern indicates that some of the CipA3 molecules are bound to both Cel8A and Cel9B. It should be noted that in the lanes containing cohesin-enzyme combinations the antibody staining is much more intense, which may indicate that only a proportion of the enzymes, when not bound to CipA, migrate into the non-denaturing gels.

### Enzymatic activity of the heterologous mini-cellulosomes secreted by *C. acetobutylicum*

To assess the activity of the heterologously expressed enzymes alone and in complex with mini-scaffoldin we assayed the activity of the early stationary phase culture supernatants (OD600 ~2.0) against cellohexaose (Cel9B, Cel8A and Cel9K) at 60°C, using HPAEC-PAD to monitor product release, and also 4-nitrophenyl-β-d-cellobioside (4-NPC) in the case of Cel9K. The data show that supernatants from wild-type *C. acetobutylicum* and cells expressing CipA3 alone display no detectable activity against the oligosaccharide or 4-NPC (Table 1). In contrast, cells expressing Cel8A from the thlOID promoter, either alone or with CipA3, display significant activity (Table 1), although the presence of the scaffoldin reduced the observed specific activity ~2.5-fold. The activity of Cel8A expressed only from the upstream thiolase promoter was 20-fold lower than when transcribed from thlOID (Table 1), consistent with the differential expression of the scaffoldin variants under control of one or two promoters (Figure 4). The activity for all Cel9B-expressing cell supernatants was above background, but much lower than for Cel8A. Similarly, Cel9K activity was low but was clearly above background against both cellohexaose and 4-NP.

**Table 1:** Activity of the heterologously expressed enzymes against cellohexaose and 4-NPC

**Supernatant of recombinant *C. acetobutylicum *strains**	**Substrate**	**Activity (units/mg total protein)**
Wild-type *C. aceto*	Cellohexaose	ND^a^
Cel8A	Cellohexaose	1.1 ±0.06
Cel9B	Cellohexaose	0.02 ±0.005
thlOID^b^_CipA3	Cellohexaose	ND
thlOID_Cel8A	Cellohexaose	26.9 ±4.8
thlOID_Cel9B	Cellohexaose	0.02 ±0.008
thlOID_Cel8A_CipA3	Cellohexaose	10.8 ±2.2
thlOID_Cel9B_CipA3	Cellohexaose	0.02 ±0.006
thlOID_Cel9B_Cel8A_CipA3	Cellohexaose	0.30 ±0.10
Cel9K	Cellohexaose	0.005 ±0.0001
Cel9K	4-NPC^c^	0.0005 ±0.0002
Wild-type *C. aceto*	4-NPC	ND

^a^ND: no activity detected.

^b^thlOID: genes under control of the upstream *thl* and *thl*OID promoters.

^c^4-NPC: 4-nitrophenyl-β-d-cellobioside.

## Discussion

The development of ACE technology [21] has made it possible to overcome the challenge of stably modifying the genome of *C. acetobutylicum*, and our results described here demonstrate the secretion, assembly and activity of mini-cellulosomes expressed from the chromosome of stable recombinant *C. acetobutylicum* strains, a major milestone in the engineering of this organism for butanol production via CBP. However, there are still several obstacles to be overcome. The first is the apparent impact of the gene arrangement within an operon on the expression of individual genes. As seen here and reported previously [28,29], the configuration of an operon greatly affects the expression of its genes. The rearrangement of genes within an operon results in changes in the sequence of the encoded mRNA and thus its secondary structure and its related properties. These changes will affect the rate of transcription and translation and the stability and/or degradation rate of the newly arranged mRNA molecules, ultimately resulting in altered protein levels. While we could potentially adjust the composition of the operon to identify the optimal configuration, it may be also possible to use multiple promoters to drive the expression of each component individually. The use of mono-cistronic expression constructs should also avoid a series of variations encountered when using operons. The use of BB2-standard components would easily provide assembly of cassettes containing the enzymes and/or scaffoldin under the control of different promoters. The use of promoters with different strengths may also allow a finer level of control over the proportions of each component in the secreted cellulosomes.

Another significant problem is the toxicity of heterologous cellulases when expressed in *C. acetobutylicum*[19]. The catalytic activity of the enzymes is unlikely to contribute to their lethality. A more likely explanation is that the secretion of the heterologous enzymes becomes stalled, and the resultant “blockage” causes a general defect in protein secretion leading to cell death. Thus, there is an urgent need to test this hypothesis and, if shown to be correct, approaches will need to be developed that ensure efficient export of the cellulases, possibly through the use of chaperones and appropriate secretion signals.

The third obstacle is to identify the cellulosome composition required for *C. acetobutylicum* growth on lignocellulose**.** The ability of cellulosomal hydrolases with different activities to act in synergy has been demonstrated; a trifunctional complex containing an exoglucanase (Cel48F), endoglucanase (Cel9G) and xylanase (XynZ) was shown to display greatly improved activity over bifunctional complexes [30]. While we have demonstrated the expression of both Cel8A and Cel9B, the recombinant strains developed are still unable to grow on a natural substrate, namely milled wheat straw (data not shown). Although *C. acetobutylicum* displays a small level of xylanase activity, it is possible that this is not enough to efficiently degrade the hemicellulose component of wheat straw and it may thus be necessary to introduce a xylanase as part of the enzyme cohort. Additionally, in order to best take advantage of the potential for enzyme synergy afforded by the use of a cellulosome, it may be important to also include an exo-acting cellulase, such as Cel9K, in addition to an endoglucanase (Cel8A) and endoprocessive cellulase (Cel9B). Recently, Anderson et al. [31] created a strain of *B. subtilis* able to grow on untreated natural feedstocks through expression of *C. cellulolyticum* Cel5A, Cel9E, and Cel48F, an endoglucanase/xylanase, endoglucanase/exoglucanase, and a processive endoglucanase respectively, in combination with a cell wall-associated mini-scaffoldin. When grown on pretreated feedstocks, the rate of hydrolysis by *Bacillus* cells expressing three enzymes with the scaffoldin was greater than that of those expressing only two enzymes, with expression of only one enzyme giving the weakest activity. Finally, a fourth challenge may be the apparent difficulty of expressing longer scaffoldin variants. While we have demonstrated that the shorter scaffoldin variants (CipA2 and CipA3) are readily expressed, the expression levels of the longer variants are greatly reduced. While former studies [17] have examined expression of mini-scaffoldin constructs in *C. acetobutylicum*, we are unaware of any attempted expressions of full-length heterologous scaffoldins in *C. acetobutylicum* to date. We hypothesise that there may be an issue with mRNA stability [32], either due to the length of the construct, or the high level of homology between the different scaffoldin domains. Although degradation of lignocellulose has been shown to be possible with minicellulosomes containing only three enzymes [31], should we wish to increase the complexity of our minicellulosomes in the future, we may have to examine ways of increasing the stability, for example, by examining the effects of different 5′ mRNA leader sequences, or by engineering the sequence to reduce homology.

## Conclusions

We have successfully demonstrated the expression, secretion and complex formation of three *C. thermocellum*-derived cellulosome components inserted into the genome of *C. acetobutylicum*, providing a proof of principle of the utility of the ACE method to create stable *C. acetobutylicum* strains for the expression of a functional cellulosome. Generation of a strain able to utilise lignocellulose will require further improvement of the expression levels of the cellulolytic enzymes along with increased range of catalytic activities.

## Methods

### Design of synthetic DNA fragments

All DNA fragments were designed as standard, interchangeable “parts” based on BioBrick BB-2 RFC[12] (BB2) idempotent assembly proposed by Tom Knight [33]. This assembly means that any newly composed part will adhere to its assembly standard and can be used in future assemblies [http://dspace.mit.edu/handle/1721.1/45139]. Each part consists of a DNA fragment flanked by restriction sites, called prefix at the 5′ end and suffix at the 3′ end. The BB2 prefix contains the *Eco*RI, *Not*I and *Spe*I restriction sites while the prefix contains the *Nhe*I/*Not*I/*Pst*I sites. These sites allow parts to be assembled together, forming a new part which will maintain the same prefix and suffix and contain a scar (GCTAGT, corresponding to alanine and serine) where the cut and re-ligated restriction sites (*Spe*I and *Nhe*I) were fused together. We chose to use this method to build a range of synthetic cellulosomal units of various complexities.

The full length (5.6 kb) *cipA* gene [Cthe_3077] encoding the scaffoldin protein from *C. thermocellum* was codon optimised for usage in *Clostridium acetobutylicum* and the gene synthetically constructed by DNA 2.0 (Menlo Park, CA) as a single DNA fragment lacking the stop codon. To this fragment, the ribosome binding site of the strong constitutive thiolase promoter (P*thl*) from *C. acetobutylicum* and the standard BioBrick2 prefix and suffix restriction sites were added, resulting in the BB2prefix_RBS_CipA_BB2suffix standard assembly part [deposited at GenBank, KC560744]. In parallel, the eleven scaffoldin subunits of the full length CipA, each encoding one of the nine cohesin modules, the carbohydrate binding module (CBM3a), or the X module, and flanked by the linker regions (where appropriate), were also synthesised by DNA 2.0 as standard BioBrick2 modular parts [deposited at GenBank: KC560733, KC560734, KC560735, KC560736, KC560737, KC560738, KC560739, KC560740, KC560741, KC560742, KC560743]. A list of plasmids containing all synthetic genes synthesised in BB2 format can be found in Additional file 2: Table S1.

This modular design allowed the standard assembly of a series of scaffoldin proteins with various numbers of cohesin modules and the reconstruction of the full length *cipA* [CipABB2, Genbank KC560745]. A list of plasmids created by BB2 assembly is listed in Additional file 2. As the assembly using the BB2 technology results in an alanine-serine (Ala-Ser) scar, the ten “breaking points” within the scaffoldin sequence were chosen to coincide either with an alanine (A) or a threonine (T) in the linker regions (in one case inside the second cohesin domain); the adjacent amino acid was omitted and, as a result of assembly, replaced by the serine (Ser) from the scar (The CipA amino acid sequence with highlighted features, including the BB2 assembly points can be found in Additional file 1).

The same design principles were applied to create all synthetic DNA fragments encoding the four GHs from *C. thermocellum* (Cel8A-Cthe0269, Cel9B-Cthe0543, Cel48S_Cthe2089 and Cel9K_ Cthe0412), except these genes were not codon optimised. The BB2 prefix and RBS from the *thl* promoter were inserted upstream of the coding sequence and the BB2 suffix inserted downstream resulting in standard BB2 parts (BB2prefix_RBS_GH _BB2suffix).

The *thl*OID promoter, containing the *lac* operator sequence was designed as a BB2 standard assembly part, containing the *lac* operator sequence and lacking the original RBS of the promoter sequence (The sequence of the *thl*OID promoter can be found in Additional file 1).

### Plasmid construction

All synthetic CipA scaffoldin fragments synthesised by DNA 2.0 were delivered in one of the pJ20x plasmids [Additional file 2: Table S1]. All four cellulases (Cel8A, Cel9B, Cel9K and Cel48S) were synthesised as standard BB2 parts by Biomatik (Canada) and delivered in one of the company’s in-house vectors (pBMH) [Additional file 2: Table S1] The pJ202_Flag_2xSTOP vector, carrying the Flag tag and the stop codon, was created by annealing the Flag-Stop_Fw_CCCGGGGAATTCGCGGCCGCACTAGTGATTACAAGGATGACGACGATAAGTAATGAGCTAGCGCGGCCGCCTGCAGCCCGGG and Flag-Stop_Rev_CCCGGGCTGCAGGCGGCCGCGCTAGCTCATTACTTATCGTCGTCATCCTTGTAATCACTAGTGCGGCCGCGAATTCCCCGGG primers and inserting the *Eco*RI/*Pst*I-digested fragment into the pJ202 backbone. All three pJ20x plasmid backbones were used for Biobrick2 standard assembly to create all desired DNA fragments for integration into the *C. acetobutylicum* genome [list of BB2 plasmids in Additional file 2].

The native CipA gene was amplified from the genomic DNA of *C. thermocellum* using the following primers: CipAtherm_Fw CAC*CATATG*AGAAAAGTCATCAGTATGCTCTTAGTTGTGGC and CipAtherm_Rev CAC*ACTAGT*CTGTGCGTCGTAATCACTTGATG. Several cloning steps were necessary to create the native CipA variant in order to introduce the same RBS and Flag tag as the synthetic CipA genes. Firstly, the 5.6 kb PCR product was digested with *Nde*I/*Eco*RI and *Spe*I restriction enzymes (Fermentas) and two smaller fragments (a 4.5 kb and a 1.1 kb) were gel purified. The larger 4.5 kb fragment was ligated into an *Eco*RI/*Spe*I-cut BB2 backbone which carried the Flag tag (pJ202_Flag_2×Stop), generating the pJ202_truncCipA_Flagx2Stop plasmid. The truncated, Flag-tagged CipA was released from this plasmid by *Eco*RI/*Nhe*I digest and ligated together with the previously gel purified smaller (1.1 kb) PCR fragment into *Nde*I/*Nhe*I-digested pMTL_JH16_RBS, resulting in thepMTL_JH16_RBS_nativeCipA_Flag plasmid.

All BB2-assembled DNA fragments (synthetic and native *cipA* variants and all four cellulases) were fused at the carboxy-terminal with the Flag epitope tag (added as a BB2 fragment) and subsequently cloned as a *Not*I/*Nhe*I fragment into the pMTL-JH16 integration vector [21], resulting in pMTL-JH16_RBS_gene integration vectors [Additional file 2: Table S2]. Integration vectors which contained an additional thiolase promoter (P*thl*) upstream of the CipA coding regions were generated by inserting the *thl* promoter into the pMTL-JH16_CipAx vectors as a *Not*I-*Nde*I fragment with the *Nde*I site overlapping the CipA start codon.

In order to achieve sufficient repression in *E. coli* cells of constructs containing cellulases and cellulosomal operons driven by the *thl*OID promoter, we modified the pJ201 Biobrick2 vector and the pMTL-JH16 integration vector to contain the *lacI*^*Q*^ gene. The *lacI*^*Q*^ gene sequence was amplified from the pGEX-6P-1 (GE Heathcare) vector using the FwlacIQ CCCGGGGTTTAAACGACACCATCGAATGGTGCAAAACC and RevlacIQ CCCGGGGTTTAAACTTAATTGCGTTGCGCTCACTGC primers and subsequently cloned into the *Sma*I site of pJ201 and the *Pme*I site of the pMTL-JH16, resulting in the pJ201_lacIQ and pMTL-JH16_lacIQ plasmids [Additional file 2: Table S2].

### Bacterial strains

*Escherichia coli* TOP10 (Invitrogen) strain was used for all BioBrick 2 cloning steps. *E. coli* cultures were grown in Luria-Bertani (LB) broth at 37°C with rotary shaking at 200 rpm, or on LB plates supplemented with the appropriate antibiotics (ampicillin 100 μg/ml, kanamycin 50 μg/ml, zeocin 25 μg/ml, chloramphenicol 25 μg/ml). The same *E. coli* strain containing the pAN2 [34] plasmid was used for *in vivo* methylation of plasmid DNA prior to *Clostridium acetobutylicum* ATCC 824 transformations.

*E. coli* BL21 (DE3) (NEB) cells were used for cloning most of the cellulases and cellulosomal operons behind the *thl*OID promoter. For cloning the more complex cellulosomal operons (*thl*OID_Cel9B_CipA3 and *thl*OID_Cel9B_Cel8A_CipA3) into the modified integration vector (pMTL-JH16_lacIQ), *E. coli* XL10-Gold KanR ultracompetent cells (Agilent) were used.

BL21 and XL10 *E. coli* cells with LacI^Q^-containing vectors were all grown at 30°C in order to further reduce the toxicity of the enzymes – this required an extra 24 h growth on plates and an extra 6 h in liquid culture.

*Clostridium acetobutylicum* ATCC 824 wild type strain and the subsequent recombinant strains [Additional file 2: Table S2] were grown in static culture at 37°C under an anaerobic atmosphere of N2:H2:CO2 (80:10:10, vol:vol:vol) in an anaerobic workstation (Don Whitley, UK) using 2×YTG media prereduced overnight under the same conditions. Methylated plasmids were transferred into *C. acetobutylicum* by electroporation as described previously [35]. Recombinant strains were selected on antibiotic-containing CGM plates [36] (primary transformants on 15 μg/ml thiamphenicol and double recombinants on 40 μg/ml erythromycin) and screened for chromosomal integration by PCR as described previously [21].

*Clostridium thermocellum* ATCC 27405 was grown at 60°C in air tight serum bottles containing pre-reduced GS-2 media [37] and used for preparation of genomic DNA (Blood and tissue DNA extraction kit, Qiagen) for subsequent use as a template for the *cipA* gene.

### Culture conditions, SDS-PAGE and western blot analysis

Recombinant *C. acetobutylicum* strains expressing the CipA variants were grown overnight in 30 ml liquid 2YTGmedia (pH 5.6) until late exponential phase (OD600 > 3). The cells were harvested by centrifugation (4°C for 10 minutes at 7000×*g*) and the culture supernatant incubated for one hour with 40 mg Avicel (Sigma). Proteins exhibiting affinity for cellulose were recovered by centrifugation (4°C for 10 minutes at 10000×*g*), mixing the cellulose pellet with SDS-PAGE loading buffer (100× less than the starting overnight culture), and boiling for 10 minutes. Samples were centrifuged briefly (1 minute at 13000×*g*) and analysed by SDS-PAGE on NuPAGE® Novex 4-12% precast polyacrylamide gels (Invitrogen), followed by western blot analysis using ANTI-FLAG M2 monoclonal antibody-horseradish peroxidase conjugate (Sigma) following the suppliers recommendation.

Initially, recombinant *C. acetobutylicum* strains expressing the GHs (Cel8A, Cel9B, Cel9K and Cel48S) on their own were grown overnight until late exponential phase (OD600 > 3) in buffered 2YTG (40 mM MOPS, pH 7). Growing the cultures until late exponential phase resulted in higher protein yield but more degraded products. In later experiments, the recombinant strains expressing the GHs driven by the modified *thl*OID promoter and the cellulosomal operons were grown in buffered 2YTG (40 mM MOPS, pH 7) until early exponential phase (OD600 around 0.7), before the pH of the cultures dropped below 6. Buffering the culture media and analysing the strains at an early exponential phase was necessary as we have observed that the heterologously expressed cellulases were unstable in the acidic conditions created by the host. 10 ml starter cultures were inoculated with fresh colonies grown on erythromycin (40 μg/ml) containing GCM plates. From this neat culture, three 10-fold serial dilutions were prepared. After growth overnight in the anaerobic cabinet at 37°C, starter cultures showing an early exponential phase of growth were used to inoculate 30 ml buffered 2×YTG to an OD600 of 0.05. At an OD600 around 0.7 (a pH of 6.0 – 6.5), the cultures were harvested by centrifugation at 4°C for 10 m at 7000×*g*. Supernatants were collected and processed for SDS denaturing or native PAGE analysis. For SDS-PAGE analysis, the supernatants were concentrated 50 or 100× by DOC-TCA precipitation (final concentration of 0.03% DOC and 10% TCA) as described by Schwarz et al. [38].

Concentrated samples were analysed on NuPAGE® Novex 4-12% Bis-Tris PAGE precast polyacrylamide gels (Invitrogen), followed by western blot analysis using ANTI-FLAG M2 monoclonal antibody-horseradish peroxidise conjugate (1:3000, Sigma) following the suppliers’ recommendations.

### Non-denaturing PAGE

Non-denaturing PAGE analysis was used to assess *in vivo* mini-cellulosome complex formation. All culture supernatants were treated by addition of proteinase inhibitor cocktail (Calbiochem Proteinase Inhibitor Cocktail Kit VII) at a dilution ratio of 50:1 and concentration of the supernatant by around 70 to 80-fold via centrifugation using Corning® Spin-X® UF concentrators (6 ml, 10000 MWCO) at 4000 rpm in a swing bucket rotor. 10 μl of the concentrated samples were mixed with the same volume of Novex Tris-Glycine Native Sample Buffer (2X) (Invitrogen) and samples were loaded onto a NuPAGE® Novex 3-8% Tris-Acetate Gel (Invitrogen) and run using Novex® Tris-Glycine Native Running Buffer (Invitrogen) at 150 V for 2 hours. Transfer and western blot analysis were carried out according to the standard protocol using ANTI-FLAG M2 monoclonal antibody-horseradish peroxidise conjugate (1:3000 dilution, Sigma), following the supplier’s recommendation.

### Enzyme assays

To measure enzyme activity, the supernatants from early stationary phase cultures (O.D.600 ~2.0) were first dialysed in to 100 mM MES buffer, pH 6.5 (Buffer A). Heterologously expressed Cel9B, Cel8A and Cel9K were assayed by measuring the degradation of cellohexaose (0.5 mM) at 60°C in Buffer A. Samples were taken at time points and boiled to stop the reaction before the total products were analysed by high performance anion-exchange chromatography with pulsed amperometric detection (HPAEC-PAD; Dionex). The assay was performed in triplicate and initial rates of product formation determined for each construct. Cel9K was also assayed using 4-nitrophenyl-β-d-cellobioside as the substrate (4-NPC). The reaction, which was carried out at 60°C for up to 40 min contained 4-NPC in Buffer A, and the product, 4-nitrophenol, was monitored continuously at 400 nm and quantified using a ϵ_400 nm_ of 7500 M^-1^ cm^-1^. Total cellular protein was determined by Bradford assay on sonicated cell pellets that had been resuspended in Buffer A. Using this value specific activity for each construct was calculated as units of product released per mg of total cellular protein (1 unit = 1 μmol/min).

## Abbreviations

CBM: Carbohydrate-binding module; GH: Glycoside hydrolase; CMC: Carboxymethyl cellulose; CGM: Clostridial growth medium; SDS-PAGE: Sodium dodecyl sulfate polyacrylamide gel electrophoresis.

## Competing interests

The authors declare that they have no competing interests.

## Authors’ contributions

JTH, KW and NPM designed and coordinated the study, KK and BJW carried out the experiments, analysed the results and wrote the paper, KS helped with the experimental work, AJ and DNB carried out and analysed the enzyme activity assays. All authors read and approved the final manuscript.

## Supplementary Material

Additional file 1 **Amino acid and nucleotide sequences.** This file contains the amino acid sequence of the *C. thermocellum* CipA protein with highlighted BioBrick 2 assembly points and the nucleotide sequence of the *thl*OID promoter in BioBrick 2 format. Click here for file

Additional file 2 **Plasmids and bacterial strains used in this study.** This file contains two tables and a list of BioBrick 2 plasmids created by using the synthetic fragments described in Table S1. Table S1 summarizes the plasmids containing the BioBrick 2 fragments synthesised and Table S2 describes the heterologous *C. acetobutylicum* strains expressing *C. thermocellum* derived cellulosomal subunits and operons and the plasmids used to create these strains. Click here for file

## References

[B1] Kumar M Gayen K Developments in biobutanol production: new insights Applied Energy 2011 88 1999 2012 10.1016/j.apenergy.2010.12.055

[B2] Olson DG McBride JE Joe Shaw A Lynd LR Recent progress in consolidated bioprocessing Curr Opin Biotechnol 2012 23 396 405 10.1016/j.copbio.2011.11.026 22176748

[B3] Grange D Haan R Zyl W Engineering cellulolytic ability into bioprocessing organisms Appl Microbiol Biotechnol 2010 87 1195 1208 10.1007/s00253-010-2660-x 20508932

[B4] Argyros DA Tripathi SA Barrett TF Rogers SR Feinberg LF Olson DG Foden JM Miller BB Lynd LR Hogsett DA Caiazza NC High ethanol titers from cellulose by using metabolically engineered thermophilic, anaerobic microbes Appl Environ Microbiol 2011 77 8288 8294 10.1128/AEM.00646-11 21965408 PMC3233045

[B5] Yamada R Taniguchi N Tanaka T Ogino C Fukuda H Kondo A Direct ethanol production from cellulosic materials using a diploid strain of *Saccharomyces cerevisiae* with optimized cellulase expression Biotechnol Biofuels 2011 4 8 10.1186/1754-6834-4-8 21496218 PMC3095537

[B6] Chang J-J Ho F-J Ho C-Y Wu Y-C Hou Y-H Huang C-C Shih M-C Li W-H Assembling a cellulase cocktail and a cellodextrin transporter into a yeast host for CBP ethanol production Biotechnol Biofuels 2013 6 19 10.1186/1754-6834-6-19 23374631 PMC3599373

[B7] Kumar M Goyal Y Sarkar A Gayen K Comparative economic assessment of ABE fermentation based on cellulosic and non-cellulosic feedstocks Appl Energy 2012 93 193 204

[B8] Servinsky MD Kiel JT Dupuy NF Sund CJ Transcriptional analysis of differential carbohydrate utilization by *Clostridium acetobutylicum* Microbiology 2010 156 3478 3491 10.1099/mic.0.037085-0 20656779

[B9] Fierobe H-P Mingardon F Chanal A Engineering cellulase activity into *Clostridium acetobutylicum* Methods Enzymol 2012 510 301 16 22608733 10.1016/B978-0-12-415931-0.00016-1

[B10] Lütke-Eversloh T Bahl H Metabolic engineering of *Clostridium acetobutylicum*: recent advances to improve butanol production Curr Opin Biotechnol 2011 22 634 647 10.1016/j.copbio.2011.01.011 21377350

[B11] Lee SF Forsberg CW Rattray JB Purification and characterization of Two Endoxylanases from *Clostridium acetobutylicum* ATCC 824 Appl Environ Microbiol 1987 53 644 650 16347312 10.1128/aem.53.4.644-650.1987 PMC203729

[B12] López-Contreras AM Gabor K Martens AA Renckens BAM Claassen PAM van der Oost J de Vos WM Substrate-induced production and secretion of cellulases by *Clostridium acetobutylicum* Appl Environ Microbiol 2004 70 5238 5243 10.1128/AEM.70.9.5238-5243.2004 15345405 PMC520844

[B13] Sabathé F Soucaille P Characterization of the CipA scaffolding protein and in vivo production of a minicellulosome in *clostridium acetobutylicum* J Bacteriol 2003 185 1092 1096 10.1128/JB.185.3.1092-1096.2003 12533485 PMC142813

[B14] Bayer EA Belaich J-P Shoham Y Lamed R The cellulosomes: multienzyme machines for degradation of plant cell wall polysaccharides Annu Rev Microbiol 2004 58 521 554 10.1146/annurev.micro.57.030502.091022 15487947

[B15] Fontes CMGA Gilbert HJ Cellulosomes: highly efficient nanomachines designed to deconstruct plant cell wall complex carbohydrates Annu Rev Biochem 2010 79 655 681 10.1146/annurev-biochem-091208-085603 20373916

[B16] Hall J Ali S Surani MA Hazlewood GP Clark AJ Simons JP Hirst BH Gilbert HJ Manipulation of the repertoire of digestive enzymes secreted into the gastrointestinal tract of transgenic mice Nat Biotechnol 1993 11 376 379 10.1038/nbt0393-376 7763439

[B17] Perret S Casalot L Fierobe H-P Tardif C Sabathe F Belaich J-P Belaich A Production of Heterologous and Chimeric Scaffoldins by *Clostridium acetobutylicum* ATCC 824 J Bacteriol 2004 186 253 257 10.1128/JB.186.1.253-257.2004 14679247 PMC303433

[B18] Mingardon F Perret S Bélaïch A Tardif C Bélaïch J-P Fierobe H-P Heterologous production, assembly, and secretion of a minicellulosome by *Clostridium acetobutylicum* ATCC 824 Appl Environ Microbiol 2005 71 1215 1222 10.1128/AEM.71.3.1215-1222.2005 15746321 PMC1065181

[B19] Mingardon F Chanal A Tardif C Fierobe H-P The issue of secretion in heterologous expression of *Clostridium cellulolyticum* cellulase-encoding genes in *Clostridium acetobutylicum* ATCC 824 Appl Environ Microbiol 2011 77 2831 2838 10.1128/AEM.03012-10 21378034 PMC3126403

[B20] Chanal A Mingardon F Bauzan M Tardif C Fierobe H-P Scaffoldin modules serving as “Cargo” domains to promote the secretion of heterologous cellulosomal cellulases by *Clostridium acetobutylicum* Appl Environ Microbiol 2011 77 6277 6280 10.1128/AEM.00758-11 21764966 PMC3165433

[B21] Heap JT Ehsaan M Cooksley CM Ng Y-K Cartman ST Winzer K Minton NP Integration of DNA into bacterial chromosomes from plasmids without a counter-selection marker Nucleic Acids Res 2012 40 8 e59 10.1093/nar/gkr1321 22259038 PMC3333862

[B22] Olson DG Giannone RJ Hettich RL Lynd LR Role of the CipA scaffoldin protein in cellulose solubilization, as determined by targeted gene deletion and complementation in *Clostridium thermocellum* J Bacteriol 2013 195 733 739 10.1128/JB.02014-12 23204466 PMC3562095

[B23] Lamed R Kenig R Morag E Yaron S Shoham Y Bayer E Nonproteolytic cleavage of aspartyl proline bonds in the cellulosomal scaffoldin subunit from *Clostridium thermocellum* Appl Biochem Biotechnol 2001 90 67 73 10.1385/ABAB:90:1:67 11257808

[B24] Raman B Pan C Hurst GB Rodriguez M Jr McKeown CK Lankford PK Samatova NF Mielenz JR Impact of pretreated switchgrass and biomass carbohydrates on *Clostridium thermocellum* ATCC 27405 cellulosome composition: a quantitative proteomic analysis PLoS ONE 2009 4 e5271 10.1371/journal.pone.0005271 19384422 PMC2668762

[B25] Zverlov VV Schwarz WH Bacterial cellulose hydrolysis in anaerobic environmental subsystems—*Clostridium thermocellum* and *Clostridium stercorarium*, thermophilic plant-fiber degraders Ann NY Acad Sci 2008 1125 298 307 10.1196/annals.1419.008 18378600

[B26] Carvalho AL Dias FM Nagy T Prates JA Proctor MR Smith N Bayer EA Davies GJ Ferreira LM Romao MJ Fontes CMGA Gilbert HJ Evidence for a dual binding mode of dockerin modules to cohesins Proc Natl Acad Sci USA 2007 104 3089 3094 10.1073/pnas.0611173104 17360613 PMC1805526

[B27] Carvalho AL Dias FM Prates JA Nagy T Gilbert HJ Davies GJ Ferreira LM Romao MJ Fontes CM Cellulosome assembly revealed by the crystal structure of the cohesin-dockerin complex Proc Natl Acad Sci USA 2003 100 13809 13814 10.1073/pnas.1936124100 14623971 PMC283503

[B28] Lim HN Lee Y Hussein R Fundamental relationship between operon organization and gene expression Proc Natl Acad Sci USA 2011 108 10626 10631 10.1073/pnas.1105692108 21670266 PMC3127940

[B29] Temme K Zhao D Voigt CA Refactoring the nitrogen fixation gene cluster from *Klebsiella oxytoca* Proc Natl Acad Sci USA 2012 109 7085 7090 10.1073/pnas.1120788109 22509035 PMC3345007

[B30] Fierobe H-P Mingardon F Mechaly A Bélaïch A Rincon MT Pagès S Lamed R Tardif C Bélaïch J-P Bayer EA Action of designer cellulosomes on homogeneous versus complex substrates: controlled incorporation of three distinct enzymes into a defined trifunctional scaffoldin J Biol Chem 2005 280 16325 16334 10.1074/jbc.M414449200 15705576

[B31] Anderson TD Miller JI Fierobe H-P Clubb RT Recombinant *Bacillus subtilis* that grows on untreated plant biomass Appl Environ Microbiol 2013 79 867 876 10.1128/AEM.02433-12 23183968 PMC3568581

[B32] Carrier TA Keasling JD Controlling messenger RNA stability in bacteria: strategies for engineering gene expression Biotechnol Prog 1997 13 699 708 10.1021/bp970095h 9413129

[B33] Knight T Idempotent Vector Design for Standard Assembly of Biobricks MIT Synthetic Biology Working Group Technical Reports 2003 http://hdl.handle.net/1721.1/21168

[B34] Heap JT Pennington OJ Cartman ST Carter GP Minton NP The ClosTron: a universal gene knock-out system for the genus *Clostridium* J Microbiol Methods 2007 70 452 464 10.1016/j.mimet.2007.05.021 17658189

[B35] Heap JT Kuehne SA Ehsaan M Cartman ST Cooksley CM Scott JC Minton NP The ClosTron: mutagenesis in *Clostridium* refined and streamlined J Microbiol Methods 2010 80 49 55 10.1016/j.mimet.2009.10.018 19891996

[B36] Hartmanis MGN Gatenbeck S Intermediary metabolism in *Clostridium acetobutylicum*: levels of enzymes involved in the formation of acetate and butyrate Appl Environ Microbiol 1984 47 1277 1283 16346566 10.1128/aem.47.6.1277-1283.1984 PMC240219

[B37] Johnson EA Madia A Demain AL Chemically defined minimal medium for growth of the anaerobic cellulolytic thermophile *Clostridium thermocellum* Appl Environ Microbiol 1981 41 1060 1062 16345748 10.1128/aem.41.4.1060-1062.1981 PMC243859

[B38] Schwarz K Fiedler T Fischer R-J Bahl H A Standard Operating Procedure (SOP) for the preparation of intra- and extracellular proteins of *Clostridium acetobutylicum* for proteome analysis J Microbiol Methods 2007 68 396 402 10.1016/j.mimet.2006.09.018 17098314

