# Supplementary material for: Secretion and assembly of functional mini-cellulosomes from synthetic chromosomal operons in Clostridium acetobutylicum ATCC 824
Source: Biotechnol Biofuels. 2013 Aug 20;6:117. doi: 10.1186/1754-6834-6-117 (PMC3765823; doi:10.1186/1754-6834-6-117)
Supplement: Additional file 1 — Amino acid and nucleotide sequences. This file contains the amino acid sequence of the C. thermocellum CipA protein with highlighted BioBrick 2 assembly points and the nucleotide sequence of the thlOID promoter in BioBrick 2 format. [file 1754-6834-6-117-S1.doc]

**CipA protein sequence [UniProt: Q06851]**

**MRKVISMLLVVAMLTTIFAAMIPQTVSAATMTVEIGKVTAAVGSKVEIPITLKGVPSKGMANCDFVLGYDPNVLEVTEVKPGSIIKDPDPSKSFDSAIYPDRKMIVFLFAEDSGRGTYAITQDGVFATIVATVKSAAAAPITLLEVGAFADNDLVEISTTFVAGGVNLGSSVPTTQPNVPSDGVVVEIGKVTGSVGTTVEIPVYFRGVPSKGIANCDFVFRYDPNVLEIIGIDPGDIIVDPNPTKSFDTAIYPDRKIIVFLFAEDSGTGAYAITKDGVFAKIRATVKSSAPGYITFDEVGGFADNDLVEQKVSFIDGGVNVGNATPTKGATPTNTATPTKSATATPTRPSVPTNTPTNTPANTPVSGNLKVEFYNSNPSDTTNSINPQFKVTNTGSSAIDLSKLTLRYYYTVDGQKDQTFWCDHAAIIGSNGSYNGITSNVKGTFVKMSSSTNNADTYLEISFTGGTLEPGAHVQIQGRFAKNDWSNYTQSNDYSFKSASQFVEWDQVTAYLNGVLVWGKEPGGSVVPSTQPVTTPPATTKPPATTKPPATTIPPSDDPNAIKIKVDTVNAKPGDTVNIPVRFSGIPSKGIANCDFVYSYDPNVLEIIEIKPGELIVDPNPDKSFDTAVYPDRKIIVFLFAEDSGTGAYAITKDGVFATIVAKVKSGAPNGLSVIKFVEVGGFANNDLVEQRTQFFDGGVNVGDTTVPTTPTTPVTTPTDDSNAVRIKVDTVNAKPGDTVRIPVRFSGIPSKGIANCDFVYSYDPNVLEIIEIEPGDIIVDPNPDKSFDTAVYPDRKIIVFLFAEDSGTGAYAITKDGVFATIVAKVKSGAPNGLSVIKFVEVGGFANNDLVEQKTQFFDGGVNVGDTTEPATPTTPVTTPTTTDDLDAVRIKVDTVNAKPGDTVRIPVRFSGIPSKGIANCDFVYSYDPNVLEIIEIEPGDIIVDPNPDKSFDTAVYPDRKIIVFLFAEDSGTGAYAITKDGVFATIVAKVKSGAPNGLSVIKFVEVGGFANNDLVEQKTQFFDGGVNVGDTTEPATPTTPVTTPTTTDDLDAVRIKVDTVNAKPGDTVRIPVRFSGIPSKGIANCDFVYSYDPNVLEIIEIEPGDIIVDPNPDKSFDTAVYPDRKIIVFLFAEDSGTGAYAITKDGVFATIVAKVKEGAPNGLSVIKFVEVGGFANNDLVEQKTQFFDGGVNVGDTTEPATPTTPVTTPTTTDDLDAVRIKVDTVNAKPGDTVRIPVRFSGIPSKGIANCDFVYSYDPNVLEIIEIEPGELIVDPNPTKSFDTAVYPDRKMIVFLFAEDSGTGAYAITEDGVFATIVAKVKSGAPNGLSVIKFVEVGGFANNDLVEQKTQFFDGGVNVGDTTEPATPTTPVTTPTTTDDLDAVRIKVDTVNAKPGDTVRIPVRFSGIPSKGIANCDFVYSYDPNVLEIIEIEPGDIIVDPNPDKSFDTAVYPDRKIIVFLFAEDSGTGAYAITKDGVFATIVAKVKEGAPNGLSVIKFVEVGGFANNDLVEQKTQFFDGGVNVGDTTVPTTSPTTTPPEPTITPNKLTLKIGRAEGRPGDTVEIPVNLYGVPQKGIASGDFVVSYDPNVLEIIEIEPGELIVDPNPTKSFDTAVYPDRKMIVFLFAEDSGTGAYAITEDGVFATIVAKVKEGAPEGFSAIEISEFGAFADNDLVEVETDLINGGVLVTNKPVIEGYKVSGYILPDFSFDATVAPLVKAGFKVEIVGTELYAVTDANGYFEITGVPANASGYTLKISRATYLDRVIANVVVTGDTSVSTSQAPIMMWVGDIVKDNSINLLDVAEVIRCFNATKGSANYVEELDINRNGAINMQDIMIVHKHFGATSSDYDAQ**

**Signal peptide**

**Cohesin domains**

**CBM3 (carbohydrate binding module)**

**Pro/Thr-rich Linkers**

**Non-proteolytic cleavage sites within the second cohesin domain (Mapped by Lamed et al. [18])**

**BB2 assembly “Breaking points”; the underlined sequences were replaced by AS in the final BB2 assembled full length CipA (CipA_BB2)**

**X domain**

**Type II dockerin domain**

***thl*OID promoter**

**BB2 prefix -35 -10 lac operator**

**GAATTCGCGGCCGCACTAGT**tttttaacaaaatata**ttgata**aaaataataatagtggg**tataat**taaTGTGGAATTGTGAGCGCTCACAATTGGTGTgttgttagagaaaacgtataaattagggataaactatggaacttatgaaatagattgaaatggtttatctgt**GCTAGCGCGGCCGCTGCAG**

**BB2 suffix**
