# Supplementary material for: Secretion and assembly of functional mini-cellulosomes from synthetic chromosomal operons in Clostridium acetobutylicum ATCC 824
Source: Biotechnol Biofuels. 2013 Aug 20;6:117. doi: 10.1186/1754-6834-6-117 (PMC3765823; doi:10.1186/1754-6834-6-117)
Supplement: Additional file 2 — Plasmids and bacterial strains used in this study. This file contains two tables and a list of BioBrick 2 plasmids created by using the synthetic fragments described in Table S1. Table S1 summarizes the plasmids containing the BioBrick 2 fragments synthesised and Table S2 describes the heterologous C. acetobutylicum strains expressing C. thermocellum derived cellulosomal subunits and operons and the plasmids used to create these strains. [file 1754-6834-6-117-S2.doc]

**Table S1 Plasmids containing synthetic BioBrick 2 fragments**.

| **Plasmid number** | **Plasmid name** | **GenBank accession of the BB2 fragments/genes** | **Plasmid source** | **Selection marker/ origin of replication** |
| --- | --- | --- | --- | --- |
|  | pJ201_RBS_SPcoh1 | KC560733 | DNA 2.0 (USA) | Kanamycin/pUC |
|  | pJ204_Coh2 L7 | KC560734 | DNA 2.0 (USA) | Ampicillin/pUC |
|  | pJ202_L33 CBM3a L26 | KC560735 | DNA 2.0 (USA) | Zeocin/pUC |
|  | pJ201_L8 Coh3 L4 | KC560736 | DNA 2.0 (USA) | Kanamycin /pUC |
|  | pJ204_L13 Coh4 L5 | KC560737 | DNA 2.0 (USA) | Ampicillin /pUC |
|  | pJ202_L15 Coh5 L5 | KC560738 | DNA 2.0 (USA) | Zeocin/pUC |
|  | pJ201_L15 Coh6 L5 | KC560739 | DNA 2.0 (USA) | Kanamycin/pUC |
|  | pJ204L15 Coh7 L5 | KC560740 | DNA 2.0 (USA) | Ampicillin /pUC |
|  | pJ202_L15 Coh8 L6 | KC560741 | DNA 2.0 (USA) | Zeocin/pUC |
|  | pJ201_L13 Coh9 x | KC560742 | DNA 2.0 (USA) | Kanamycin/pUC |
|  | pJ204_x and dockerin | KC560743 | DNA 2.0 (USA) | Ampicillin/pUC |
|  | pJ201_DNA2.0_CipA | KC560744 | DNA 2.0 (USA) | Kanamycin/pUC |
|  | pBMH_Cel9B_Cthe_0543 | X60545 | Biomatik (Canada) | Ampicillin /pUC |
|  | pBMH_Cel9K_Cthe_0412 | AF039030 | Biomatik (Canada) | Ampicillin /pUC |
|  | pBMH_Cel48S_Cthe_2089 | L06942 | Biomatik (Canada) | Ampicillin /pUC |
|  | pBMH_Cel8A_Cthe_0269 | K03088 | Biomatik (Canada) | Ampicillin /pUC |
|  | pBMH_thlOID | Promoter sequence available in Additional file 1 | Biomatik (Canada) | Ampicillin /pUC |

**List of BioBrick 2 plasmids created by using the synthetic fragments from Table 1.**

The numbers following the pJ20x backbones correspond to the plasmid numbers listed in Table1 ( i.e pJ202_1+2 = pJ202_RBS_SPcoh1+ Coh2 L7)

1. pJ202_1+2
2. pJ204_3+4
3. pJ201_5+6
4. pJ202_7+8
5. pJ204_9+10
6. pJ201_9+10+11
7. pJ201_1+2+3+4
8. pJ204_5+6+7+8
9. pJ202_1+2+3+4+5+6+7+8
10. pJ204_1+2+3+4+5+6+7+8+9+10+11 = CipA without stop codon or Flag tag (BB2_CipA)
11. pJ202**_BB2_CipAfull_Flag**
12. pJ204_1+2+3 =CipA2
13. pJ202**_1+2+3 =CipA2_Flag**
14. pJ202_1+2+3+4 =CipA3
15. pJ202_**1+2+3+4 =CipA3_Flag**
16. pJ201_1+2+3+4+5 = CipA4
17. pJ202_**1+2+3+4+5 = CipA4_Flag**
18. pJ204_1+2+3+4+5+6 =CipA5
19. pJ202_**1+2+3+4+5+6 =CipA5_Flag**
20. pJ202_1+2+3+4+5+6+7 =CipA6
21. pJ202_**1+2+3+4+5+6+7 =CipA6_Flag**
22. pJ201_1+2+3+4+5+6+7+8 = CipA7
23. pJ202_**1+2+3+4+5+6+7+8 = CipA7_Flag**
24. pJ204_1+2+3+4+5+6+7+8+9 =CipA8
25. pJ202**_1+2+3+4+5+6+7+8+9 =CipA8_Flag**
26. pJ202_1+2+3+4+5+6+7+8+9+10 =CipA9
27. pJ202_**1+2+3+4+5+6+7+8+9+10 =CipA9_Flag**

**List of additional BioBrick 2 plasmids created**

1. pJ202_Flagx2STOP
2. pJ202_truncCipA_Flagx2Stop
3. pJ202_**DNA2.0_CipA full_Flag**
4. pJ202_**Cel8A_Flag**
5. pJ202_**Cel9B_Flag**
6. pJ202_**Cel48S_Flag**
7. pJ202_**Cel9K_Flag**
8. pJ202_**thlOID Cel8A_Flag**
9. pJ202_**thlOID Cel9B_Flag**
10. pJ201_lac_thlOID
11. pJ201_lac_**thlOID_Cel9B_CipA3**
12. pJ201_lac_**thlOID_Cel8A_CipA3**
13. pJ201_Cel9B_Cel8A_CipA3
14. pJ201_lac_**thlOID_Cel9B_Cel8A_CipA3**
15. pJ201_lac_**thlOID_CipA3**

All fragments in **Bold** were also subcloned in either pMTL-JH16 or pMTL-JH16_lac plasmids, resulting the integration vectors listed in **Table 2.**

**Table S2 Heterologous *C. acetobutylicum* strains expressing *C. thermocellum* derived cellulosomal subunits and operons.**

All Flag-tagged heterologous genes/operons were subcloned into the NotI/ NheI site of the pMTL-JH16 [15] or pMTL-JH16_lac vectors to create the integration vectors listed below. These vectors allowed the insertion of the heterologous genes into the thiolase locus of the host.

| **Strain** | **Integration vector used to create the strain** | **Phenotype of the heterologous strains** |
| --- | --- | --- |
| Cac_CipA2 | pMTL-JH16_RBS_CipA2_Flag | *C. acetobutylicum* strain expressing a mini-scaffoldin with two type I cohesin domains and a CBM3a (from the chromosomal *thl* promoter) |
| Cac_CipA3 | pMTL-JH16_RBS_CipA3_Flag | *C. acetobutylicum* strain expressing a mini-scaffoldin with three type I cohesin domains and a CBM3a (from the chromosomal *thl* promoter) |
| Cac_CipA4 | pMTL-JH16_RBS_CipA4_Flag | *C. acetobutylicum* strain expressing a mini-scaffoldin with four type I cohesin domains and a CBM3a from the chromosomal *thl* promoter |
| Cac_CipA5 | pMTL-JH16_RBS_CipA5_Flag | *C. acetobutylicum* strain expressing a mini-scaffoldin with five type I cohesin domains and a CBM3a from the chromosomal *thl* promoter |
| Cac_CipA6 | pMTL-JH16_RBS_CipA6_Flag | *C. acetobutylicum* strain expressing a mini-scaffoldin with six type I cohesin domains and a CBM3a from the chromosomal *thl* promoter |
| Cac_CipA7 | pMTL-JH16_RBS_CipA7_Flag | *C. acetobutylicum* strain expressing a mini-scaffoldin with seven type I cohesin domains and a CBM3a from the chromosomal *thl* promoter |
| Cac_CipA8 | pMTL-JH16_RBS_CipA8_Flag | *C. acetobutylicum* strain expressing a mini-scaffoldin with eight type I cohesin domains and a CBM3a from the chromosomal *thl* promoter |
| Cac_CipA9 | pMTL-JH16_RBS_CipA9_Flag | *C. acetobutylicum* strain expressing a mini-scaffoldin with nine type I cohesin domains and a CBM3a from the chromosomal *thl* promoter |
| Cac_ CipABB2 | pMTL-JH16_RBS_CipABB2_full_Flag | *C. acetobutylicum* strain expressing the full length CipA scaffoldin protein built by BB2 from the chromosomal *thl* promoter |
| Cac_CipADNA2.0 | pMTL-JH16_RBS_CipADNA2.0_full_Flag | *C. acetobutylicum* strain expressing the full length CipA scaffoldin protein synthesised by DNA 2.0 from the chromosomal *thl* promoter |
| Cac_CipAnative | pMTL-JH16_RBS_CipAnative_full_Flag | *C. acetobutylicum* strain expressing the full length native CipA scaffoldin protein amplified from *C. thermocellums* genomic DNA from the chromosomal *thl* promoter |
| Cac_thl_CipA2 | pMTL-JH16_thl_CipA2_Flag | *C. acetobutylicum* strain expressing a mini-scaffoldin with two type I cohesin domains and a CBM3a inserted into the *thl* locus and driven by an additional *thl* promter |
| Cac_thl_CipA3 | pMTL-JH16_thl_CipA3_Flag | *C. acetobutylicum* strain expressing a mini-scaffoldin with three type I cohesin domains and a CBM3a inserted into the *thl* locus and driven by an additional *thl* promter |
| Cac_thl_CipABB2 | pMTL-JH16_thl_CipABB2_full_Flag | *C. acetobutylicum* strain expressing the full length CipA scaffoldin protein built by BB2 standard assembly inserted into the *thl* locus and driven by an additional *thl* promter |
| Cac_thl_CipADNA2.0 | pMTL-JH16_thl_CipADNA2.0_full_Flag | *C. acetobutylicum* strain expressing the full length CipA scaffoldin protein synthesised by DNA 2.0 inserted into the *thl* locus and driven by an additional *thl* promter |
| Cac_ Cel8A | pMTL-JH16_RBS_Cel8A_Flag | *C. acetobutylicum* strain expressing Cel8A from the chromosomal *thl* promoter |
| Cac_ Cel9B | pMTL-JH16_RBS_Cel9B_Flag | *C. acetobutylicum* strain expressing Cel9B from the chromosomal *thl* promoter |
| Cac_ Cel9K | pMTL-JH16_RBS_Cel9K_Flag | *C. acetobutylicum* strain expressing Cel9K from the chromosomal *thl* promoter |
| Cac_ Cel48S | pMTL-JH16_RBS_Cel48S_Flag | *C. acetobutylicum* strain expressing Cel48S from the chromosomal *thl* promoter |
| Cac_ thlOID_Cel8A | pMTL-JH16_lac_thlOID_ Cel8A_Flag | *C. acetobutylicum* strain expressing Cel8A inserted into the *thl* locus and driven by an additional lac repressed *thl* promter |
| Cac_ thlOID_Cel9B | pMTL-JH16_lac_thlOID_Cel9B_Flag | *C. acetobutylicum* strain expressing Cel9B inserted into the *thl* locus and driven by an additional lac repressed *thl* promter |
| Cac_ thlOid_CipA3 | pMTL-JH16_lac_thlOid_CipA3_Flag | *C. acetobutylicum* strain expressing a mini-scaffoldin with three type I cohesin domains and a CBM3a inserted into the *thl* locus and driven by an additional lac repressed *thl* promter |
| Cac_ thlOid_Cel8A_CipA3 | pMTL-JH16_lac_thlOid_Cel8A_Flag_CipA3_Flag | *C. acetobutylicum* strain expressing a unifunctional minicellulosome with Cel8A activity and with a mini-scaffoldin with three type I cohesin domains and a CBM3a driven by an additional lac repressed *thl* promter |
| Cac_ thlOid_Cel9B_CipA3 | pMTL-JH16_lac_thlOid_Cel9B_Flag_CipA3_Flag | *C. acetobutylicum* strain expressing a unifunctional minicellulosome with Cel9B activity and with a mini-scaffoldin with three type I cohesin domains and a CBM3a driven by an additional lac repressed *thl* promter |
| Cac_ thlOid_Cel9B_Cel8A_CipA3 | pMTL-JH16_lac_thlOid_Cel9B_Flag_Cel8A_Flag_CipA3_Flag | *C. acetobutylicum* strain expressing a bifunctional minicellulosome with Cel9B and Cel8A activities and with a mini-scaffoldin with three type I cohesin domains and a CBM3a driven by an additional lac repressed *thl* promter |
